# Preparation, Thermal Stability, and Preliminary Gas Separation Performance of Furan-Based Bio-Polyimide Films

**DOI:** 10.3390/polym17101362

**Published:** 2025-05-16

**Authors:** Wei Jiao, Jie Zhou, Qinying Gu, Zijun Liu, Jiashu Pan, Jiangchun Qin, Yiyi Zhu, Dengbang Jiang, Jiayang Hu

**Affiliations:** 1 National and Local Joint Engineering Research Center for Green Preparation Technology of Biobased Materials, Yunnan Minzu University, Kunming 650500, China; ajiao10151015@163.com (W.J.); 18988442351@163.com (J.Z.); z3047ly3550wjk@163.com (Q.G.); liuzijun06@163.com (Z.L.); 18609660811@163.com (J.P.); 18311848105@163.com (J.Q.); 18787388469@163.com (Y.Z.); 2 Hubei Academy of Forestry, Wuhan 430075, China

**Keywords:** bio-based polyimides, furan, films, gas separation, mechanical properties, thermal stability, CO_2_ separation, CO_2_/N_2_ selectivity

## Abstract

The need for renewable alternatives to petroleum-based polymers is growing in response to environmental concerns and resource depletion. Polyimides (PIs), which are traditionally synthesized from petroleum-derived monomers, raise sustainability issues. In this work, renewable 2,5-furandicarboxylic acid (FDCA) was employed as a sustainable feedstock to synthesize a bio-based diamine monomer, N,N′-bis(4-aminophenyl)furan-2,5-dicarboxamide (FPA). Subsequently, FPA was polymerized with various aromatic dianhydrides through thermal imidization, yielding four distinct bio-based polyimide (FPA-PI) films. The resulting films exhibited exceptional thermal stability, with 5% weight loss temperatures exceeding 425 °C and char yields ranging from 54% to 60%. Mechanical characterization revealed high elastic moduli (2.14–3.20 GPa), moderate tensile strengths (50–99 MPa), and favorable aging resistance. Gas permeation tests demonstrated promising CO_2_/N_2_ separation performance, with FPA-DODDA achieving superior CO_2_/N_2_ selectivity (27.721) compared to commercial films such as Matrimid^®^, polysulfone, and polycarbonate, while FPA-BPFLDA exhibited enhanced CO_2_ permeability (P(CO_2_) = 2.526 Barrer), surpassing that of Torlon^®^. The CO_2_/N_2_ separation performance of these FPA-PI films is governed synergistically by size-sieving effects and solution-diffusion mechanisms. This work not only introduces a novel synthetic route for bio-based polymers but also highlights the potential of replacing conventional petroleum-based materials with renewable alternatives in high-temperature and gas separation applications, thereby advancing environmental sustainability.

## 1. Introduction

With the increasing severity of global environmental challenges and the gradual depletion of fossil resources, the development of renewable alternatives to petroleum-based materials has emerged as a critical focus in polymer research [1,2]. Currently, approximately 99% of global plastic production relies on petroleum-derived feedstocks, with 4–6% of Europe’s annual oil consumption allocated to plastics manufacturing, of which 40% is utilized in the packaging sector [3]. This high dependence on fossil resources not only exacerbates environmental pollution and greenhouse gas emissions but also raises the long-term threat of resource depletion. Therefore, developing high-performance polymer materials based on renewable biomass resources has become a key strategy for achieving sustainable development and environmental protection [4,5].

Polyimides (PIs), as a class of high-performance polymers containing -CO-NR-CO- groups in their molecular structure, are widely used in the nuclear industry, liquid crystals, lasers, nanotechnology, microelectronics, and aerospace due to their excellent mechanical properties, dimensional stability, chemical resistance, low volatility, non-toxicity, self-extinguishing properties, and radiation stability [6,7,8]. The preparation of PIs typically employs a “two-step method”, where diamines and dianhydrides first form polyamic acid (PAA) solutions in polar aprotic solvents, followed by thermal or chemical imidization to convert to polyimides [9,10]. Traditional polyimides are mainly synthesized from petroleum-based monomers, such as 4,4′-diaminodiphenyl ether (ODA) and pyromellitic dianhydride (PMDA) [11]. However, the production of these petroleum-based monomers is usually accompanied by the emission of toxic and harmful substances and high energy consumption, which does not align with the concepts of green chemistry and sustainable development. Therefore, developing polyimide materials based on renewable biomass resources has become a research hotspot in the field of polymer materials in recent years [12,13].

Research on bio-based polyimides mainly focuses on developing renewable biomass-derived diamines and dianhydrides to replace traditional petroleum-based monomers [14]. Among them, 2,5-furandicarboxylic acid (FDCA), as an important bio-based platform compound, has been identified by the U.S. Department of Energy as one of the “high-value chemicals derived from biomass” [15]. FDCA can be synthesized from C6 sugars in lignocellulosic biomass through furan derivatives, offering advantages such as renewability, environmental friendliness, and easy accessibility [3]. In recent years, research on FDCA-based polyimides materials has made significant progress, showing potential to replace traditional petroleum-based polyimides [14,16]. For example, in the work of Ma et al. [16], two novel bio-based diamines, N,N′-bis(3-aminophenyl)furan-2,5-dicarboxamide (m-FDDA) and N,N′-bis(4-aminophenyl)furan-2,5-dicarboxamide (p-FDDA), were synthesized by incorporating renewable 2,5-furandicarboxylic acid (FDCA). Subsequently, the corresponding aromatic PIs were prepared via a two-step polycondensation process using these diamines with commercially available aromatic dianhydrides, including 3,3′,4,4′-biphenyltetracarboxylic dianhydride (BPDA), 4,4′-(hexafluoroisopropylidene)diphthalic anhydride (6FDA), and 2,2-bis [4-(3,4-dicarboxyphenoxy)phenyl]propane dianhydride (BPADA).

PIs also exhibit significant potential in gas separation applications, particularly for CO_2_ capture and separation [17,18,19]. By modifying the molecular chain packing state and free volume of PI films, a competitive balance between permeability and selectivity can be achieved [20,21]. Extensive research has been reported on petroleum-based PI films. For instance, Bei et al. [22] grafted task-specific ionic liquids (TSIL) as bulky side groups onto the PI backbone via Schiff base reactions to prepare TSIL-PI films. With 0.8 wt% TSIL loading, the CO_2_ permeability coefficient increased from 5.28 Barrer to 10.2 Barrer, while CO_2_/N_2_ selectivity improved from 21.9 to 92.8. This remarkable enhancement is attributed to the rigid TSIL side chains restricting the mobility of PI chains and packing, thereby increasing free volume to facilitate rapid CO_2_ transport. The constrained chain motion simultaneously enhanced molecular sieving effects [23], achieving synergistic improvements in both permeability and selectivity. Chuah et al. [24] incorporated 20% functionalized UiO-66-NH2 into PI to fabricate mixed matrix membranes (MMMs), denoted as UiO-66-NH2/PI MMMs. Results showed a 38% increase in CO_2_ permeability compared to pure PI, with CO_2_/N_2_ selectivity reaching 37.1, primarily due to CO_2_-specific adsorption by amino groups. Notably, research on bio-based PIs for gas separation remains in its early stages. Hu et al. [18] developed a series of Tröger’s Base (TB)-PI films using bio-based dianhydride monomers (e.g., lignin-derived compounds) and TB-containing diamine monomers. These films exhibited a CO_2_ permeability of up to 702 Barrer and exceptional thermal stability, with 5% decomposition temperatures (T_d_^5%^) exceeding 440 °C, highlighting their potential for gas separation applications. However, most diamine monomers for the synthesis of PIs still rely on petroleum-based precursors. This limitation stems from the incompatibility of aromatic diamines with microbial and plant cellular systems [25], making their direct biosynthesis challenging [26]. Consequently, despite the promising technical prospects of PI gas separation films, their environmental feasibility remains constrained by monomer sourcing issues—a critical gap that has not been adequately addressed in the existing literature. Moreover, even the currently reported bio-based diamine monomers for PIs synthesis predominantly originate from biomass resources rather than fully sustainable pathways [1].

**Scheme 1:**
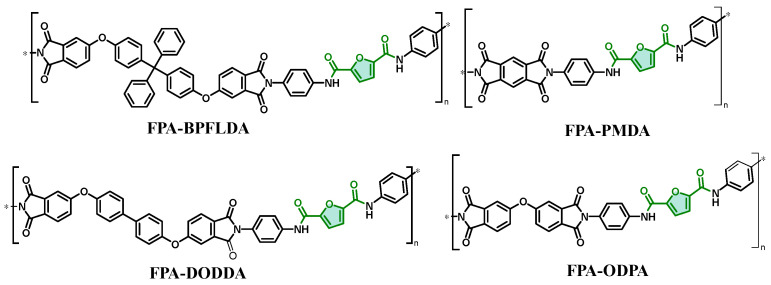
Bio-based PIs synthesized in this work (green shading: biobased feedstock origin; *: indicates simulated chain scission sites for repeating unit visualization).

**Scheme 2:**
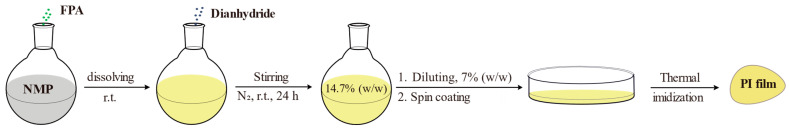
Synthetic scheme for FPA-PI films.

This work builds upon previous work by Ma et al. [16] to broaden the investigation of furan-based bio-polyimides. Starting from bio-based 2,5-furandicarboxylic acid (FDCA) as the raw material, a bio-based diamine monomer, N,N′-bis(4-aminophenyl)furan-2,5-dicarboxamide, was synthesized. To simplify the nomenclature, this monomer is abbreviated as FPA in this study (replacing the original designation “p-FDDA”). Subsequently, the diamine monomer FPA was polymerized with various aromatic dianhydrides (e.g., ODPA, BPFLDA) to prepare a series of polyamic acid (PAA) precursor solutions. Through thermal imidization, four FPA-PI films were successfully expanded (Scheme 1 and Scheme 2), and their mechanical properties, thermal stability, and gas separation performance were characterized.

## 2. Materials and Methods

### 2.1. Materials

2,5-Furandicarboxylic acid (FDCA, 99%), 4-nitroaniline (99%), 4-nitrophthalonitrile (98%), 4,4′-sulfonyldiphenol (SDP, 99.8%), 4,4′-biphenol (DOD, 99%), 4,4′-(hexafluoroisopropylidene)diphenol (BPAF, 98%), 9,9-bis(4-hydroxyphenyl)fluorene (BPFL, 98%), 1,4-benzenediol (HQ, 98%), and dianhydride monomers pyromellitic dianhydride (PMDA, 98%), 3,3′,4,4′-benzophenonetetracarboxylic dianhydride (BTDA, 98%), 4,4′-oxydiphthalic anhydride (ODPA, 98%), palladium on carbon (Pd/C, 5%), N,N-dimethylformamide (DMF, 99.5%), and N-methyl-2-pyrrolidone (NMP, 99.5%) were purchased from Adamas-beta^®^ (Shanghai Titan Technology Co., Ltd., Shanghai, China). Xylene (99.0%) was purchased from Tianjin Zhiyuan Chemical Reagent Co., Ltd. (Tianjin, China); concentrated hydrochloric acid (36–38%) from Chongqing Chuandong Chemical (Group) Co., Ltd. (Chongqing, China); acetic anhydride (Ac_2_O, 98.5%), hydrazine monohydrate (N_2_H_4_·H_2_O, 80%), oxalyl chloride ((COCl)_2_, 98.5%), methanol (MeOH, 99.5%), and anhydrous potassium hydroxide (KOH, 99.8%) from Chengdu Kelong Chemical Co., Ltd. (Chengdu, China); dichloromethane (DCM, 99.5%) from Yunnan Four Leaf Clover Biotechnology Co., Ltd. (Yunnan, China); and tetrahydrofuran (THF, 99.5%) was purchased from Greagent-reagent^®^ (Shanghai Titan Technology Co., Ltd., Shanghai, China). NMP was dried over 4 Å molecular sieves for 48 h before use; DCM and THF were refluxed with calcium hydride for 5 h; and other reagents were used as received without further purification. The ether-containing dianhydride was synthesized by modifying the method of Hsiao et al. [27].

### 2.2. Synthesis of FDCDCl

**Scheme 3:**
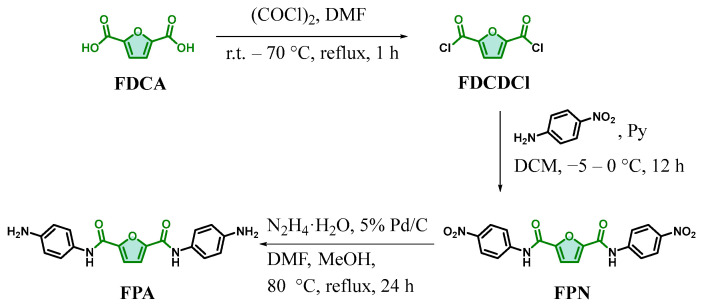
Synthesis route of FPA.

The synthesis route for the diamine monomer FPA is illustrated in Scheme 3. Starting from bio-based 2,5-furandicarboxylic acid (FDCA) as the initial raw material, the bio-based diamine monomer N,N′-bis(4-aminophenyl)furan-2,5-dicarboxamide (FPA) was synthesized through reactions with 4-nitroaniline, involving acyl chlorination, amidation, and nitro group hydrogenation reduction steps.

Synthesis of 2,5-furandicarbonyl dichloride (FDCDCl). 2,5-Furandicarboxylic acid (60.00 g, 384.40 mmol) and DMF (0.2 mL) were added to a 500 mL round-bottom flask equipped with a magnetic stirrer and a condenser. Oxalyl chloride (120 mL) was slowly added dropwise with stirring. After complete addition, the mixture was heated to 70 °C and refluxed until the reaction system became clear (reflux time: 1 h). The reaction was then cooled to room temperature, and residual solvents were removed under reduced pressure. The resulting pale brown needle-like solid was sealed and stored in a refrigerator (72.76 g, yield = 99%). M. p. = 78–79 °C; ^1^H NMR (400 MHz, Chloroform-*d*) δ 7.55 (s, 2H); ^13^C NMR (101 MHz, Chloroform-*d*) δ 156.01, 149.42, 123.48; IR (KBr, ν, cm^−1^): 3143, 3110 (C-H, furan stretching), 1745, (C=O, stretching), 1564 (C=C, furan stretching), 1200 (C-O-C, stretching), 540 (C-Cl, stretching).

### 2.3. Synthesis of FPN

Synthesis of N,N′-bis(4-nitrophenyl)furan-2,5-dicarboxamide (FPN). 4-Nitroaniline (33.98 g, 245.80 mmol), pyridine (21.39 g, 270.38 mmol), and anhydrous THF (369 mL) were added to a 1 L three-necked round-bottom flask equipped with a mechanical stirrer. The mixture was cooled to −5 to 0 °C, and a solution of FDCDCl (23.59 g, 122.90 mmol) in anhydrous DCM (123 mL) was slowly added dropwise under stirring. After complete addition, the reaction was stirred at room temperature for 12 h and monitored by thin-layer chromatography (TLC; eluent: MeOH/DCM = 5%, *v*/*v*; R_f_ = 0.5). The solid was collected by filtration, slurried twice in 500 mL of deionized water, washed three times with 250 mL methanol, and dried in a blast drying oven at 80 °C to afford a light green powdery solid (45.57 g, yield = 93%). M. p. = 375.4 °C (DSC); ^1^H NMR (400 MHz, DMSO-*d*_6_) δ 10.87 (d, *J* = 3.8 Hz, 2H), 8.31 (d, *J* = 8.9 Hz, 4H), 8.06 (d, *J* = 8.8 Hz, 4H), 7.58 (s, 2H); ^13^C NMR (101 MHz, DMSO-*d*_6_) δ 155.94, 148.13, 144.43, 142.89, 124.99, 120.07, 117.13; IR (KBr, ν, cm^−1^): 3391, (N-H, amide stretching), 3119/3081 (C-H, furan/aromatic stretching), 1700, (C=O, stretching), 1610, 1500 (C=C, aromatic stretching), 1538 (-NO_2_, asym. stretching), 1331 (-NO_2_, sym. stretching), 1305 (C-N, amide stretching), 848 (C-H, aromatic p-disubst. bending).

### 2.4. Synthesis of FPA

Synthesis of N,N′-bis(4-aminophenyl)furan-2,5-dicarboxamide (FPA). A mixture of FPN (39.63 g, 100 mmol), DMF (200 mL), MeOH (200 mL), Pd/C (5% *w*/*w*, 8.51 g), and N_2_H_4_·H_2_O (60 mL) was added to a 1 L three-necked round-bottom flask equipped with a magnetic stirrer. The reaction was heated to 80 °C under a nitrogen (N_2_) atmosphere and refluxed for 24 h, with progress monitored by TLC (eluent: MeOH/DCM = 5%, *v*/*v*; R_f_ = 0.2). The mixture was filtered hot through celite, and the Pd/C catalyst was washed twice with DMF (30 mL). The filtrate was collected, and the product was precipitated by adding 1.5 L of deionized water under vigorous stirring. The resulting solid was isolated by filtration, washed three times with water, and dried at 80 °C for 48 h to afford a pale yellow powdery solid (30.83 g, yield = 92%). M. p. = 150.3 °C (DSC); ^1^H NMR (400 MHz, DMSO-*d*_6_) δ 9.95 (s, 2H), 7.45–7.30 (m, 4H), 7.28 (s, 2H), 6.78–6.41 (m, 4H), 5.07 (s, 4H); ^13^C NMR (101 MHz, DMSO-*d*_6_) δ 155.12, 148.38, 146.03, 126.40, 122.86, 115.30, 113.87. IR (KBr, ν, cm^−1^): 3435/3321, (-NH_2_, asym./sym. stretching), 3387, (N-H, amide stretching), 3115/3078 (C-H, furan/aromatic stretching), 1646, (C=O, stretching), 1608, 1515 (C=C, aromatic stretching), 1563/1327 (-NO_2_, asym./sym. stretching), 1291 (C-N, amide stretching), 826 (C-H, aromatic p-disubst. bending).

### 2.5. Synthesis of Tetranitriles

**Scheme 4:**
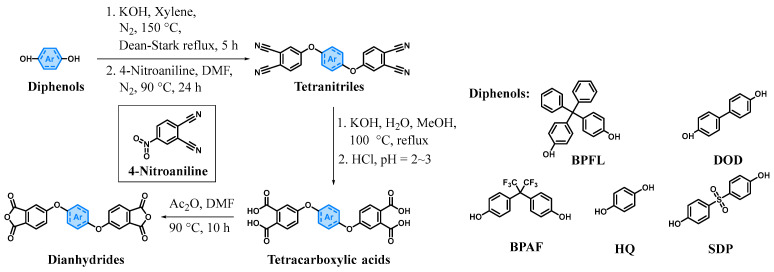
Synthesis route of dianhydrides.

**Scheme 5:**
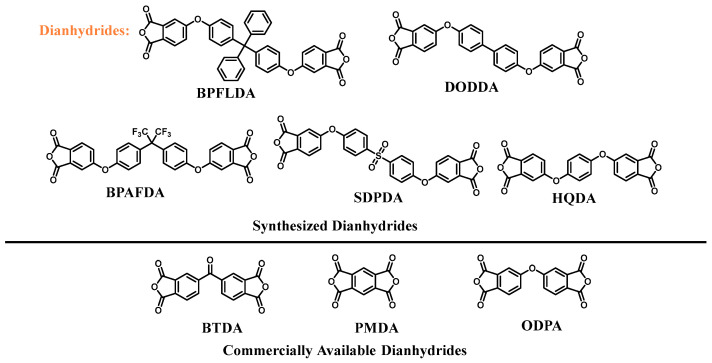
Dianhydrides synthesized in this work and commercially available dianhydrides.

The dianhydrides BTDA, PMDA, and ODPA used in this work were obtained from commercial sources. Additionally, ether-linked dianhydrides were synthesized by modifying the method reported by Hsiao et al. [27], as illustrated in Scheme 4 and Scheme 5.

Synthesis of 4,4′-[(diphenylmethylene)bis(1,4-phenyleneoxy)]diphthalonitrile (BPFLCN). BPFL (49.06 g, 140.00 mmol), anhydrous KOH (15.71 g, 280.00 mmol), and xylene (280 mL) were added to a 500 mL round-bottom flask equipped with a magnetic stirrer. The mixture was heated to 150 °C under N_2_ atmosphere and refluxed for 5 h. During the reaction, water and approximately 270 mL of xylene were removed using a Dean–Stark trap. After cooling to room temperature, 4-nitrophthalonitrile (48.48 g, 280.00 mmol) and dry DMF (280 mL) were added. The reaction mixture was heated to 90 °C under N_2_ and stirred for 24 h. Subsequently, the mixture was poured into 2 L of water under vigorous stirring. The precipitated solid was collected by filtration, washed three times with a MeOH/DCM (3:1, *v*/*v*) mixture, and vacuum-dried at 80 °C for 12 h to yield a pale yellow powdery solid (83.19 g, yield = 98%). M. p. = 269.4 °C (DSC); ^1^H NMR (400 MHz, DMSO-*d*_6_) δ 8.07 (d, *J* = 8.8 Hz, 2H), 8.01–7.91 (m, 2H), 7.79 (d, *J* = 2.6 Hz, 2H), 7.51 (d, *J* = 7.6 Hz, 2H), 7.47–7.40 (m, 2H), 7.39–7.31 (m, 4H), 7.30–7.19 (m, 4H), 7.17–6.95 (m, 4H); ^13^C NMR (101 MHz, DMSO-*d*_6_) δ 160.79, 152.61, 150.24, 142.79, 139.53, 136.34, 129.81, 128.17, 128.03, 126.07, 122.83, 122.04, 120.76, 120.15, 116.73, 115.94, 115.43, 108.29, 64.05; IR (KBr, ν, cm^−1^): 3039 (C-H, aromatic stretching), 2228 (CN, stretching), 1590, 1490 (C=C, arom. stretching), 1252 (C-O-C, stretching), 846 (C-H, aromatic p-disubst. bending).

The other tetranitrile compounds were synthesized using the same method. 4,4′-[1,4-phenylenebis(oxy)]bis(1,2-benzenedicarbonitrile) (HQCN) was synthesized from HQ (6.61 g, 60.00 mmol), anhydrous KOH (6.73 g, 120.00 mmol), and 4-nitrophthalonitrile (20.78 g, 120.00 mmol), yielding a pale yellow powdery solid (18.85 g, 87% yield). M. p. = 268–270 °C; ^1^H NMR (400 MHz, DMSO-*d*_6_) δ 8.14 (d, *J* = 8.8 Hz, 2H), 7.87 (d, *J* = 2.5 Hz, 2H), 7.52 (dd, *J* = 8.8, 2.6 Hz, 2H), 7.34 (s, 4H); ^13^C NMR (101 MHz, DMSO-*d*_6_) δ 161.28, 151.05, 136.32, 122.80, 122.35, 122.12, 116.76, 115.98, 115.49, 108.28; IR (KBr, ν, cm^−1^): 3076 (C-H, aromatic stretching), 2233 (CN, stretching), 1596, 1501 (C=C, aromatic stretching), 1244 (C-O-C, stretching), 836 (C-H, aromatic p-disubst. bending).

4,4′-[Sulfonylbis(1,4-phenyleneoxy)]bis(1,2-benzenedicarbonitrile) (SDPCN) was synthesized from SDP (35.04 g, 140.00 mmol), anhydrous KOH (15.71 g, 280.00 mmol), and 4-nitrophthalonitrile (48.48 g, 280.00 mmol), yielding a pale yellow powdery solid (66.01 g, yield = 94%). M. p. = 226.7 °C (DSC); ^1^H NMR (400 MHz, DMSO-*d*_6_) δ 8.17 (d, *J* = 8.7 Hz, 2H), 8.11–8.03 (m, 4H), 8.01 (d, *J* = 2.6 Hz, 2H), 7.60 (dd, *J* = 8.8, 2.6 Hz, 2H), 7.37 (d, *J* = 8.8 Hz, 4H); ^13^C NMR (101 MHz, DMSO-*d*_6_) δ 159.47, 159.19, 137.61, 136.94, 130.83, 125.00, 124.71, 120.63, 117.44, 116.22, 115.73, 110.46; IR (KBr, ν, cm^−1^): 3096 (C-H, aromatic stretching), 2235 (CN, stretching), 1580, 1485 (C=C, aromatic stretching), 1251 (C-O-C, stretching), 1149 (S=O, stretching),839 (C-H, p-disubst. aromatic bending), 734, 684 (C-H, trisubst. aromatic bending).

4,4′-Bis(3,4-dicyanophenoxy)biphenyl (DODCN) was synthesized from DOD (13.04 g, 70.00 mmol), anhydrous KOH (7.86 g, 140.00 mmol), and 4-nitrophthalonitrile (24.24 g, 140.00 mmol), yielding a pale brown powdery solid (27.51 g, yield = 90%). M. p. = 235–236 °C; ^1^H NMR (400 MHz, DMSO-*d*_6_) δ 8.12 (d, *J* = 8.8 Hz, 2H), 7.85 (d, *J* = 2.6 Hz, 2H), 7.81 (d, *J* = 8.5 Hz, 4H), 7.45 (dd, *J* = 8.8, 2.6 Hz, 2H), 7.30 (d, *J* = 8.5 Hz, 4H); ^13^C NMR (101 MHz, DMSO-*d*_6_) δ 160.95, 153.52, 136.63, 136.41, 128.90, 122.92, 122.25, 120.84, 116.79, 115.98, 115.47, 108.39. IR (KBr, ν, cm^−1^): 3073 (C-H, aromatic stretching), 2232 (CN, stretching), 1592, 1486 (C=C, aromatic stretching), 1251 (C-O-C, stretching), 818 (C-H, aromatic p-disubst. bending).

2,2-Bis [4-(3,4-dicyanophenoxy)phenyl]hexafluoropropane (BPAFCN) was synthesized from BPAF (47.07 g, 140.00 mmol), anhydrous KOH (15.71 g, 280.00 mmol), and 4-nitrophthalonitrile (48.48 g, 280.00 mmol), yielding a pale yellow powdery solid (82.39 g, yield = 96%). M. p. = 232–233 °C; ^1^H NMR (400 MHz, DMSO-*d*_6_) δ 8.16 (d, J = 8.7 Hz, 2H), 7.96 (d, J = 2.5 Hz, 2H), 7.54 (dd, J = 8.7, 2.6 Hz, 2H), 7.49 (d, J = 8.5 Hz, 4H), 7.36–7.24 (m, 4H); ^13^C NMR (101 MHz, DMSO-*d*_6_) δ 159.95, 155.00, 136.48, 132.13, 128.88, 128.23, 125.39, 123.71, 123.19, 122.53, 119.98, 116.92, 115.88, 115.40, 109.22, 63.41; ^19^F NMR (376 MHz, DMSO-*d*_6_) δ −63.36; IR (KBr, ν, cm^−1^): 3088 (C-H, aromatic stretching), 2236 (CN, stretching), 1588, 1508, 1485 (C=C, aromatic stretching), 1248(C-O-C, stretching), 1023(C-F, aromatic stretching), 850 (C-H, aromatic p-disubst. bending).

### 2.6. Synthesis of Tetracarboxylic Acids and Dianhydrides

Synthesis of 5,5′-[(diphenylmethanediylbis(oxy-1,4-phenylene))]bis(isobenzofuran-1,3-dione) (BPFLDA) [28]. BPFLCN (60.47 g, 100 mmol), KOH (44.89 g, 800.00 mmol), methanol (264 mL), and water (144 mL) were added to a 500 mL round-bottom flask equipped with a magnetic stirrer. The mixture was refluxed at 100 °C until ammonia evolution ceased, as monitored by litmus paper. Concentrated HCl was then added dropwise in an ice bath to adjust the pH to 2–3, precipitating a white solid. The solid was collected by filtration, slurried twice with water, and vacuum-dried at 70 °C for 24 h to yield a grayish-white blocky solid, BPFLCOH (62.62 g, yield = 92%), which was used directly in the next step without further purification or characterization.

The crude product BPFLCOH (54.45 g, 80 mmol) and anhydrous DMF (40 mL) were added to a 250 mL round-bottom flask equipped with a magnetic stirrer. The mixture was stirred at 90 °C for 10 min, followed by the addition of 160 mL acetic anhydride (Ac_2_O). Stirring was continued for 10 h, after which the mixture was cooled to room temperature and filtered. The collected solid was slurried four times in a mixed solvent of acetic anhydride/anhydrous DMF (10:1, *v*/*v*; the DMF ratio was adjusted as needed based on solubility). Residual solvents were removed in a vacuum oven at 100 °C, and the product was further dried in a forced-air oven at 190 °C to yield a white powdery solid (45.85 g, yield = 89%). M. p. = 258–259 °C; ^1^H NMR (400 MHz, DMSO-*d*_6_) δ 8.05 (d, *J* = 8.4 Hz, 2H), 7.97 (d, *J* = 7.6 Hz, 2H), 7.57–7.34 (m, 10H), 7.28 (d, *J* = 8.4 Hz, 4H), 7.12 (d, *J* = 8.3 Hz, 4H); ^13^C NMR (101 MHz, DMSO-*d*_6_) δ 163.68, 162.67, 162.51, 153.03, 150.28, 142.79, 139.55, 134.14, 129.87, 128.22, 128.06, 127.92, 126.10, 124.88, 124.59, 120.80, 120.30, 112.77, 64.10; IR (KBr, ν, cm^−1^): 3064 (C-H, aromatic stretching), 1851, (C=O, asym. stretching), 1777 (C=O, sym. stretching), 1594, 1481 (C=C, aromatic stretching), 1279 (C-O-C, stretching).

The other dianhydrides were synthesized using the same method.

Synthesis of 5,5′-(1,4-phenylenebisoxy)bis(isobenzofuran-1,3-dione) (HQDA) [29]. HQCN (7.00 g, 19.30 mmol) and KOH (8.66 g, 154.40 mmol) were reacted to yield a crude tetracarboxylic acid as a white blocky solid, HQCOH (8.24 g, yield = 97%). HQCOH (8.24 g, 18.80 mmol) was treated with Ac_2_O (38 mL) under reflux conditions. The resulting product was isolated as a white powdery solid, HQDA (6.46 g, yield = 85%). M. p. = 268.5 °C (DSC); ^1^H NMR (400 MHz, DMSO-*d*_6_) δ 8.11 (d, *J* = 8.3 Hz, 2H), 7.61 (d, *J* = 10.7 Hz, 4H), 7.36 (s, 4H); ^13^C NMR (101 MHz, DMSO-*d*_6_) δ 164.35, 163.13, 162.99, 151.91, 134.65, 128.35, 125.46, 125.15, 123.06, 113.31; IR (KBr, ν, cm^−1^): 3102, 3075 (C-H, aromatic stretching), 1847, (C=O, asym. stretching), 1771 (C=O, sym. stretching), 1601, 1481 (C=C, aromatic stretching), 1270 (C-O-C, stretching).

Synthesis of 5,5′-[sulfonylbis(oxy-1,4-phenylene)]bis(isobenzofuran-1,3-dione) (SDPDA) [30]. SDPCN (66.01 g, 131.36 mmol) and KOH (58.96 g, 1050.88 mmol) were reacted to yield a crude tetracarboxylic acid as a white blocky solid, SDPCOH (68.13 g, yield = 90%). SDPCOH (67.58 g, 116.82 mmol) was treated with Ac_2_O (230 mL) under reflux conditions. The product was isolated as a white powdery solid, SDPDA (48.23 g, yield = 76%). M. p. = 243.4 °C (DSC); ^1^H NMR (400 MHz, DMSO-*d*_6_) δ 8.13 (d, *J* = 8.3 Hz, 2H), 8.08 (d, *J* = 8.7 Hz, 4H), 7.77 (d, *J* = 2.3 Hz, 2H), 7.67 (dd, *J* = 8.4, 2.2 Hz, 2H), 7.35 (d, *J* = 8.4 Hz, 4H); ^13^C NMR (101 MHz, DMSO-*d*_6_) δ 162.47, 161.60, 159.34, 136.99, 134.28, 130.43, 128.04, 126.66, 120.02, 115.47; IR (KBr, ν, cm^−1^): 3101(C-H, aromatic stretching), 1846, (C=O, asym. stretching), 1777 (C=O, sym. stretching), 1583, 1481 (C=C, aromatic stretching), 1283 (C-O-C, stretching), 1149 (S=O, stretching).

Synthesis of 5,5′-[1,1′-biphenyl]-4,4′-diylbis(oxy)bis(isobenzofuran-1,3-dione) (DODDA) [29]. DODCN (16.44 g, 37.50 mmol) and KOH (16.83 g, 300.00 mmol) were reacted to yield a crude tetracarboxylic acid as a pale yellow powdery solid, DODCOH (19.17 g, yield = 99%). DODCOH (18.86 g, 36.70 mmol) was treated with Ac_2_O (72 mL) under reflux conditions. The product was isolated as a pale yellow powdery solid, DODDA (15.07 g, yield = 86%). M. p. = 287.7 °C (DSC); ^1^H NMR (400 MHz, DMSO-*d*_6_) δ 8.10 (d, *J* = 8.4 Hz, 2H), 7.85 (d, *J* = 8.4 Hz, 4H), 7.58 (dd, *J* = 8.4, 2.3 Hz, 2H), 7.51 (s, 2H), 7.32 (d, *J* = 8.1 Hz, 4H); ^13^C NMR (101 MHz, DMSO-*d*_6_) δ 163.82, 162.72, 162.56, 153.94, 136.62, 134.21, 129.01, 127.98, 125.02, 124.82, 120.93, 112.87; IR (KBr, ν, cm^−1^): 3101, 3078 (C-H, aromatic stretching), 1848, (C=O, asym. stretching), 1775 (C=O, sym. stretching), 1595, 1481 (C=C, aromatic stretching), 1280 (C-O-C, stretching).

Synthesis of 5,5′-[(hexafluoropropane-2,2-diylbis(4,1-phenyleneoxy))bis(oxy)]bis(isobenzofuran-1,3-dione) (BPAFDA) [30]. BPAFCN (78.20 g, 132.89 mmol) and KOH (59.65 g, 1063.10 mmol) were reacted to yield a crude tetracarboxylic acid as a reddish-brown blocky solid, BPAFCOH (87.95 g, yield = 99%). BPAFCOH (87.95 g, 132.36 mmol) was treated with acetic anhydride (Ac_2_O, 260 mL) under reflux conditions. The product was isolated as a white powdery solid, BPAFDA (61.48 g, yield = 74%). M. p. = 232.6 °C (DSC); ^1^H NMR (400 MHz, DMSO-*d*_6_) δ 8.12 (d, *J* = 8.7 Hz, 2H), 7.66 (d, *J* = 2.3 Hz, 2H), 7.65–7.60 (m, 2H), 7.52 (d, *J* = 9.6 Hz, 4H), 7.32 (d, *J* = 9.5 Hz, 4H); ^13^C NMR (101 MHz, DMSO-*d*_6_) δ 162.64, 162.56, 162.47, 155.46, 134.23, 132.14, 128.74, 128.01, 125.77, 125.62, 125.41, 122.54, 119.97, 119.01, 114.08, 63.44; ^19^F NMR (376 MHz, DMSO-*d*_6_) δ −63.34; IR (KBr, ν, cm^−1^): 3099 (C-H, aromatic stretching), 1851, (C=O, asym. stretching), 1781 (C=O, sym. stretching), 1593, 1481 (C=C, aromatic stretching), 1285 (C-O-C, stretching).

### 2.7. Preparation of FPA-PI Films

**Scheme 6:**
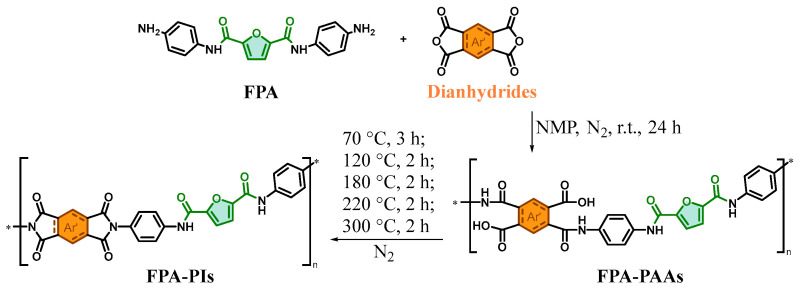
Synthesis route of FPA-PIs (green shading: biobased feedstock origin; orange shading: different segments of dianhydrides (see Scheme 5); *: indicates simulated chain scission sites for repeating unit visualization).

Bio-based polyimides were synthesized via a two-step method. First, polyamic acid (PAAs) precursors based on FPAs (FPA-PAAs) were prepared in dry NMP, followed by thermal imidization to produce bio-based PI films (FPA-PIs), as illustrated in Scheme 6.

Synthesis of PAAs (exemplified with FPA and BPFLDA). FPA (2.02 g, 6.00 mmol) and NMP (40 mL) were added to a 100 mL round-bottom flask equipped with a magnetic stirrer. BPFLDA (3.87 g, 6.00 mmol) was added under stirring, and the reaction mixture was stirred under a N_2_ atmosphere at room temperature for 24 h, yielding a viscous PAA solution with a solid content of 14.7%. Other PAA solutions were prepared analogously.

The PAA solution was diluted to a solid content of 7.0% and spin-coated onto flat-bottomed glass Petri dishes (diameter: 100 mm). A gradient heating program was employed for thermal imidization [31] to prevent defects such as pores and cracks in the film, which could be induced by rapid solvent evaporation or material decomposition due to abrupt temperature increases. The temperature protocol in this study was designed as follows: First, the sample was maintained at 70 °C under an N_2_ atmosphere with a vacuum of −0.08 MPa for 3 h to allow volatilization of the majority of NMP solvent. Subsequently, it was transferred to an oven and subjected to sequential heating steps: 120 °C for 2 h, 180 °C for 2 h, 220 °C for 2 h, and 300 °C for 2 h, with a heating rate of 2 °C·min^−1^. The timing for each step was initiated once the target temperature was attained. N_2_ purging was maintained throughout the heating process to replace ambient air gradual cooling, and the FPA-PI films were peeled off by water immersion, dried in a forced-air oven at 80 °C for 12 h, and stored under ambient conditions. The resulting bio-based PI films exhibited thicknesses of 50–70 μm.

FPA-ODPA: IR (film, ν, cm^−1^): 3337 (N-H, amide stretching), 1776, (C=O, asym. stretching), 1736 (C=O, sym. stretching), 1704 (C=O, stretching), 1370 (C-N-C, stretching), 745 (C=O, bending).

FPA-BPFLDA: IR (film, ν, cm^−1^): 3366 (N-H, amide stretching), 1776, (C=O, asym. stretching), 1730 (C=O, sym. stretching), 1667 (C=O, stretching), 1382 (C-N-C, stretching), 745 (C=O, bending)

FPA-DODDA: IR (film, ν, cm^−1^): 3334 (N-H, amide stretching), 1776, (C=O, asym. stretching), 1721 (C=O, sym. stretching), 1678 (C=O, stretching), 1379 (C-N-C, stretching), 746 (C=O, bending).

FPA-PMDA: IR (film, ν, cm^−1^): 3350 (N-H, amide stretching), 1776, (C=O, asym. stretching), 1739 (C=O, sym. stretching), 1678 (C=O, stretching), 1370 (C-N-C, stretching), 726 (C=O, bending).

### 2.8. Characterization

All NMR spectra were recorded on a Bruker AVANCE-II 400 MHz spectrometer (Bruker, Switzerland), typically with DMSO-*d*_6_, Chloroform-*d* (CDCl_3_), or D_2_O as solvents and tetramethylsilane (TMS) as an internal standard. Fourier-transform infrared (FT-IR) spectra were recorded on a Nicolet IS10 FT-IR spectrometer (Thermo Fisher Scientific, Waltham, MA, USA) in the range of 4000–400 cm^−1^ with 32 scans. Monomer precursors, diamine monomers, and dianhydride monomers were analyzed using the KBr pellet method, while PI films were directly tested. X-ray diffraction (XRD) patterns of PI films were recorded on a Bruker D8 ADVANCE A25× diffractometer (Bruker, Karlsruhe, Germany) with Cu-Kα radiation (λ = 0.15406 nm) in the 2θ range of 5–70° at a scan rate of 12°·min^−1^.

Mechanical property testing. The mechanical properties of PI films were measured using a CMT4104 computer-controlled universal testing machine (MTS SYSTEMS (CHINA) Co., Ltd., Shenzhen, China), and triplicate measurements were performed for each sample. Specimens with dimensions of 50 mm (length) × 10 mm (width) and thicknesses of 50–70 μm were tested at a tensile rate of 10 mm/min.

Thermal property testing. The glass transition temperature (Tg) of PIs was measured using a differential scanning calorimeter (DSC 214 Polyma, Netzsch, Selb, Germany). Specifically, the DSC curves of PIs were recorded during the second heating cycle. The procedure involved first weighing 5–10 mg of the sample in an aluminum crucible; then the first heating stage—heating from 20 °C to 300 °C at a rate of 10 °C·min^−1^ to eliminate thermal history; the first cooling stage—cooling from 300 °C to 20 °C at 10 °C·min^−1^; and the second heating stage—heating from 20 °C to 400 °C at 10 °C·min^−1^. All heating/cooling programs were conducted under a N_2_ atmosphere. Additionally, the melting points (M. p., °C) of some monomer precursors and monomers were determined using the same DSC instrument at a heating rate of 10 °C·min^−1^, while others were measured with a micro-melting point apparatus (X-5, Beijing Tech Instruments Co., Ltd., Beijing, China). Thermogravimetric analysis (TGA) of PIs was performed using a synchronized thermal analyzer (STA 449 F3, Netzsch, Selb, Germany) under N_2_, with a temperature range of 30 °C to 800 °C and a heating rate of 10 °C·min^−1^.

Gas permeability testing. The pure gas permeability coefficients (P, Barrer) of CO_2_ and N_2_ through PI films were measured using a GTR-721-3S gas transmission rate tester (Jinan Sike Testing Technology Co., Ltd., Jinan, China) via the pressure difference method. The permeability coefficient P is expressed in Barrer (1 Barrer = 1 × 10^−10^ cm^3^(STP)·cm·cm^−2^·s^−1^·cmHg^−1^). Tests were conducted at 23 °C under 50% relative humidity (%RH) following ASTM D3985-17 [32]. To minimize experimental errors, the thickness of each film was measured three times and averaged, and three independent samples of each polyimide film were tested. The ideal selectivity $\alpha_{ij}$ of the films was calculated using the following equation:

(1) $$\alpha_{ij}=\frac{P_{i}}{P_{j}}$$

where $P_{i}$ and $P_{j}$ represent the permeability coefficients of gas i and gas j, respectively.

## 3. Results and Discussion

### 3.1. Structural Characterization of FPA

**Figure 1:**
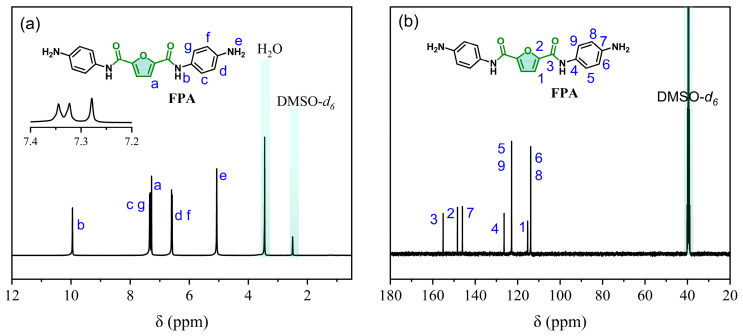
(**a**) ^1^H NMR and (**b**) ^13^C NMR spectra of FPA in DMSO-*d*_6_.

The synthesis routes of diamine monomer FPA are illustrated in Figure 1. Starting from bio-based 2,5-furandicarboxylic acid (FDCA) as the initial raw material, the bio-based diamine monomer N,N′-bis(4-aminophenyl)furan-2,5-dicarboxamide (FPA) was synthesized through reactions with 4-nitroaniline, involving acyl chlorination, amidation, and nitro group hydrogenation reduction steps. FPA’s structure was characterized by FT-IR and NMR. In the ^1^H NMR spectra (Figure 1a), the protons on the furan ring and the amino group (-NH_2_) of FPA exhibit chemical shifts at δ 7.28 ppm (labeled a) and δ 5.07 ppm (labeled e), respectively. The protons on the benzene ring of FPA resonate at δ 7.45–7.30 ppm (labeled c and g) and δ 6.78–6.41 ppm (labeled d and f). Additionally, the amide proton (-CONH-) appears at δ 9.95 ppm (labeled b). The integration ratios of all signals are consistent with the expected number of protons in the molecular structure. In the ^13^C NMR spectrum (Figure 1b), the tertiary carbon and quaternary carbon on the furan ring exhibit chemical shifts at δ 115.30 ppm (labeled 1) and δ 148.38 ppm (labeled 2), respectively. The carbonyl carbon (-C=O) in the amide group (-CONH-) resonates at δ 155.12 ppm (labeled 3), while the carbons on the benzene ring are observed at δ 146.03 ppm (labeled 7), δ 126.40 ppm (labeled 4), δ 122.86 ppm (labeled 5 and labeled 9), and δ 113.87 ppm (labeled 6 and labeled 8). Furthermore, the FT-IR spectrum (see 2.4) reveals characteristic vibrational bands: the N-H stretching of the amide group at 3387 cm^−1^, -C=O stretching at 1646 cm^−1^, asymmetric and symmetric stretching vibrations of the nitro group (-NO_2_) at 1563 cm^−1^ and 1327 cm^−1^, respectively, and aromatic -C=C stretching vibrations at 1608 cm^−1^ and 1515 cm^−1^. Collectively, these spectroscopic data (^1^H NMR, ^13^C NMR and FT-IR) provide conclusive evidence for the successful synthesis of FPA.

### 3.2. Intrinsic Viscosity of FPA-PAAs and Solubility Testing of FPA-PIs

Bio-based PIs (FPA-PIs) were synthesized via thermal imidization. Due to their limited solubility, conventional gel permeation chromatography (GPC) could not determine the molecular weights of FPA-PIs. Therefore, the intrinsic viscosities ([η]) of PAA solutions were measured using an Ubbelohde viscometer (Taizhou Jiaojiang Huan Guang Glass Instrument Co., Ltd., Taizhou, China), which reflects the hydrodynamic volume and chain extension of PAA in solution. According to the Mark–Houwink equation, molecular weight and intrinsic viscosity exhibit a positive correlation. As shown in Table 1, the intrinsic viscosities of all FPA-PAA samples ranged from 0.26 to 2.38 dL·g^−1^. Notably, when [η] fell below 0.5 dL·g^−1^, film formation was unachievable (FPA-SDPDA: 0.28 dL·g^−1^, FPA-BPAFDA: 0.26 dL·g^−1^ and FPA-BTDA: 0.49 dL·g^−1^); due to the low molecular weight of the PIs, the films become brittle or discontinuous during the thermal imidization process, resulting in the failure to form continuous films. Conversely, when [η] exceeds 0.6 dL·g^−1^, enhanced film-forming properties are achieved during the thermal imidization process (FPA-BPFLDA: 2.38 dL·g^−1^, FPA-DODDA: 0.60 dL·g^−1^, FPA-PMDA: 1.08 dL·g^−1^, FPA-ODPA: 0.93 dL·g^−1^). However, for FPA-HQDA (FPA-HQDA: 1.23 dL·g^−1^), the rigid furan backbone and amide groups (-CONH-) result in poor solubility in the solvent (NMP), resulting in microphase separation during solvent evaporation and disrupted film continuity [33], combined with localized stress imbalances during thermal processing that induce film defects; these factors ultimately prevent effective film formation. The film-forming properties of polymers are synergistically influenced by molecular weight, structural features, and solvent selection. Future work focused on enhancing the solubility and processability of PIs could involve structural modifications such as (a) incorporating fluorine-containing groups (e.g., trifluoromethyl (-CF_3_), hexafluoroisopropyl (-C(CF_3_)_2_)) to increase interchain free volume [34,35]; (b) introducing flexible alkyl/fatty chains to improve chain mobility [9,36]; or (c) copolymerization with flexible monomers (e.g., ester-containing units) to reduce chain rigidity and intermolecular interactions [14]. Additionally, solvent strategies such as cosolvent systems (e.g., N,N-dimethylacetamide (DMAC)/1,4-butyrolactone (GBL)) can be optimized by selecting solvents using Hansen solubility parameters (HSP) [37,38,39,40]. This approach helps to regulate solvent evaporation rates and enhance polymer–solvent compatibility, thereby suppressing phase separation and significantly improving film formation.

Solubility tests (as indicated by the shaded entries in Table 1, non-film-forming FPA-PIs were excluded due to the inability to prepare testable films) revealed that all bio-based FPA-PI films exhibited poor solubility, remaining insoluble even in polar aprotic solvents such as NMP, DMF, DMAC, and dimethyl sulfoxide (DMSO). This is attributed to the rigid furan structure and amide (-CONH-) groups in FPA, which persist in limiting solubility despite the incorporation of flexible ether linkages (-O-) in the dianhydrides.

### 3.3. Morphological Characterization of FPA-PI Films

**Figure 2:**
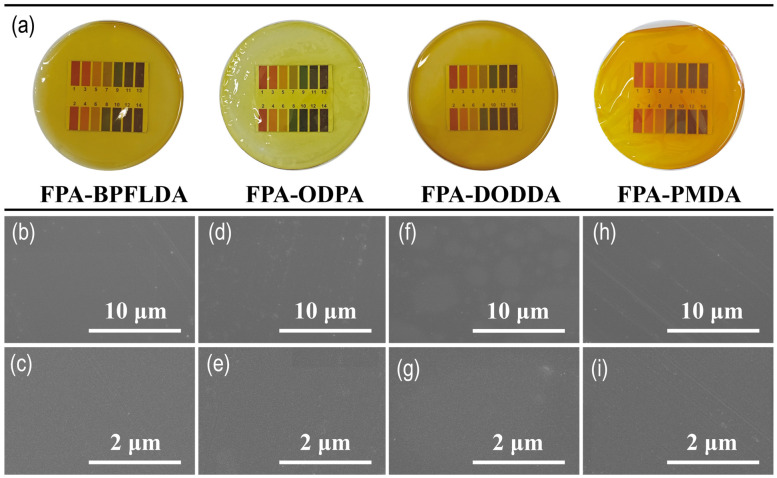
(**a**) Macroscopic morphologies and (**b**–**g**) SEM micrographs (scale bars: 10 μm and 2 μm) of FPA-PI films: (**b**,**c**) FPA-BPFLDA; (**d**,**e**) FPA-ODPA; (**f**,**g**) FPA-DODDA; and (**h**,**i**) FPA-PMDA.

As shown in Figure 2a, the macroscopic morphologies of the four bio-based FPA-PI films are presented, while the images b–j below provide their corresponding SEM micrographs with scale bars of 10 μm (20,000×) and 2 μm (100,000×). All films exhibit deep coloration, primarily attributed to the dense packing of PI chain segments within these films, which enhances light refraction [41]. Additionally, during the thermal imidization process, residual air (due to the possible presence of air not fully displaced by N_2_) may further contribute to the darkening of film color and reduced transparency. SEM analysis reveals crack-free surfaces for all films at 20,000×. However, at 100,000× magnification, while the surfaces of FPA-BPFLDA, FPA-ODPA, and FPA-PMDA remain crack-free, FPA-DODDA exhibits distinct surface cracks, which could be attributed to uneven stress distribution during the thermal imidization process [42].

### 3.4. FT-IR Characterization of FPA-PI Films

**Figure 3:**
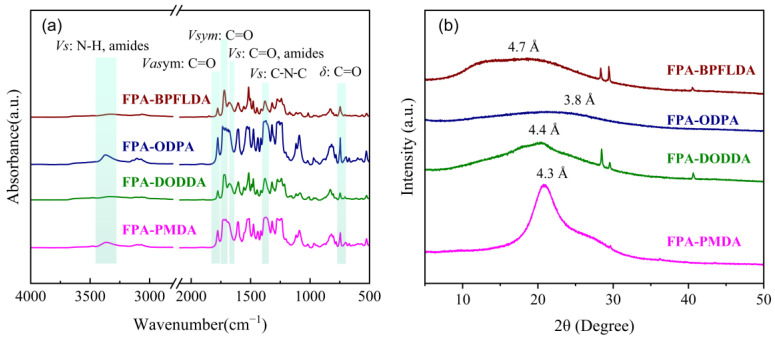
(**a**) FT-IR spectra and (**b**) XRD patterns of FPA-PIs.

The FPA-PI films were analyzed by FT-IR spectroscopy, and the results are presented in Figure 3a. The characteristic peaks of FPA-PIs are observed at approximately 1783 cm^−1^ for the asymmetric stretching vibration of the imide -C=O bond (*Vasym:* C=O), 1724 cm^−1^ for the symmetric stretching vibration of the imide -C=O bond (*Vsym:* C=O), and 1379 cm^−1^ for the stretching vibration of the imide C-N-C bond (*Vs:* C-N-C). These three peaks are the most distinctive features of polyimides [10]. Additionally, the bending vibration of the imide -C=O bond (*δ:* C=O) appears around 724 cm^−1^, serving as supplementary verification, though it may overlap with aromatic ring bending vibrations. Peaks at 1666 cm^−1^ and 3364 cm^−1^ are attributed to the stretching vibrations of the -C=O bond (*Vs:* C=O, amides) and N-H bond (*Vs:* N-H, amides) in the amide group (-CONH-), respectively. These results align well with previously reported findings for bio-based furan-containing polyimides and poly(amide-imide) systems [16,43]. The absence of carboxyl group peaks from PAA in the 3700–3400 cm^−1^ region confirms the successful synthesis of FPA-PIs [31,44].

### 3.5. XRD Characterization of FPA-PI Films

The FPA-PI films were analyzed through XRD patterns, and the results are presented in Figure 3b. The d-spacing, which typically reflects the distance between polymer chain segments, serves as an indicator of the tight packing degree of polymer chains and correlates with the free volume and gas permeability of the polymer [12,34,45]. The d-spacing was calculated by the Bragg equation λ = 2d sinθ. Distinct shoulder peaks or sharp peaks were observed near the diffraction signals of all PIs except FPA-ODPA, suggesting the presence of smaller-sized pore structures or partially ordered arrangements of PI chain segments [46]. The d-spacing values of FPA-PI films range from 3.8 to 4.7 Å, indicating generally tight chain packing. This phenomenon is attributed to the enhanced stacking of PIs molecular chains facilitated by the rigid furan rings [47]. Additionally, the structure of the dianhydride significantly influences the chain packing behavior. For example, FPA-BPFLDA (d-spacing = 4.7 Å) exhibits much larger d-spacing than FPA-ODPA (d-spacing = 3.8 Å), which is due to the steric hindrance imposed by the bulky benzene ring (-C(Ph)_2_), thereby disrupting tight chain packing.

To further explore the consistency between chain packing, d-spacing, and the density of FPA-PI films, the density (ρ) was predicted using the Synthia module in Materials Studio. This module estimates the density of amorphous polymers by calculating the ratio of the molar mass to molar volume of the repeat unit [48]. However, the predicted values in Table 1 systematically overestimate the experimental densities, as the algorithm neglects non-ideal chain arrangements caused by intermolecular interactions (e.g., van der Waals forces, hydrogen bonding) and steric hindrance effects. Despite this limitation, the relative trends in the predicted densities align with the d-spacing data, effectively reflecting differences in the tightness of chain packing. For example, FPA-ODPA exhibits the smallest d-spacing (3.8 Å) and the highest predicted density (1.391 g·cm^−3^), consistent with its tightly packed chains. In contrast, FPA-BPFLDA shows the largest d-spacing (4.7 Å) and the lowest predicted density (1.298 g·cm^−3^), corroborating the steric hindrance-induced loose packing due to its bulky benzene rings. Intermediate systems, such as FPA-DODDA (1.341 g·cm^−3^) and FPA-PMDA (1.390 g·cm^−3^), follow the same inverse correlation between d-spacing and density, which is consistent with the trends reported in the literature [45,46,49]. Additionally, an intuitive spatial ball-and-stick model of the repeat units is provided in Figure S1. These results demonstrate that the trends in d-spacing and computational density provide complementary insights into the spatial organization of polymer chains: smaller d-spacing and higher predicted density collectively indicate tighter molecular packing.

### 3.6. Thermal Property Characterization of FPA-PI Films

**Figure 4:**
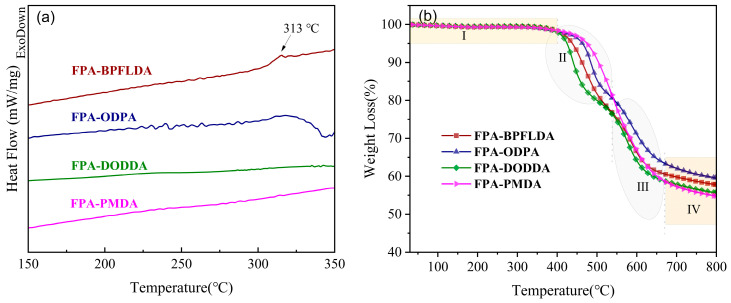
(**a**) DSC curves and (**b**) TGA curves of FPA-PI films (I: thermal stability region (<400 °C); II: furan ring pyrolysis region (425–520 °C); III: dianhydride backbone pyrolysis region (520–670 °C); IV: carbonization region (>670 °C)).

The thermal properties of four bio-based PI films were evaluated using differential scanning calorimetry (DSC). The results are shown in Figure 4a, with relevant thermal data summarized in Table 2. Notably, distinct glass transition temperatures (T_g_) were observed only for FPA-BPFLDA (T_g_ = 313 °C). For the other FPA-PIs, no distinct T_g_ was observed in the DSC curves, a feature characteristic of rigid PIs attributed to the enhanced molecular chain rigidity caused by the -CONH- groups in FPA [45]. Additionally, restricted chain segment mobility caused by interchain entanglement in PIs’ molecular chains may contribute to this phenomenon [50].

The thermal properties of four bio-based PI films were evaluated using thermogravimetric analysis (TGA). The results are shown in Figure 4b, with relevant thermal data summarized in Table 2. The thermal degradation of these four bio-based PIs can be divided into four distinct regions. Region I (<400 °C): no significant weight loss was observed for any polyimide, indicating exceptional thermal stability of the imide rings, aromatic ether linkages, and amide bonds in the polymer backbone, which underscores the high heat resistance of PIs. Region II (425–520 °C): The temperature at 5% weight loss (T_d_^5%^) exceeded 425 °C for all samples, primarily attributed to the pyrolysis of furan rings. Notably, FPA-PMDA exhibits the highest thermal stability, with its 5% weight loss temperature (T_d_^5%^) and 10% weight loss temperature (T_d_^10%^) reaching 475 °C and 504 °C, respectively. This is attributed to its rigid benzene ring structure and enhanced polymer chain packing density. Region III (520–670 °C): degradation of the dianhydride backbone dominated, driven by cleavage of aromatic ether linkages (-O-Ar-O-). Region IV (>670 °C): High-temperature carbonization occurred, with char yield from 50 to 60%. FPA-ODPA exhibited the highest char yield (59.6%), suggesting the formation of a dense carbon layer with excellent barrier effects. FPA-BPFFLDA (57.8%) followed, likely due to their high aromatic ring content. Compared to the PI-2-c reported by Ma et al. [16], PI-2-c (48%) exhibited lower char yield, which is attributed to the presence of the flexible structural group (-C(CF_3_)_2_ in 6FDA). Furthermore, the decomposition of -CF_3_ groups into volatile fluorinated molecules (such as CHF_3_) at elevated temperatures further inhibited the formation of stable carbonaceous layers in PI-2-c systems [51,52]. In summary, in this work, all FPA-PIs exhibited outstanding thermal stability (T_d_^5%^ > 425 °C), highlighting their potential for applications in the field of sustainable organic high-temperature resistant materials.

According to the US Department of Agriculture (USDA), the bio-based content in a bio-based polymer refers to the percentage of bio-based carbon in the total carbon of the polymer [10,53]. As shown in Table 2, the bio-based content of FPA-PIs ranges from 10.2% to 21.4%, with FPA-PMDA exhibiting the highest bio-based content (21.4%).

### 3.7. Mechanical Properties of FPA-PI Films

**Figure 5:**
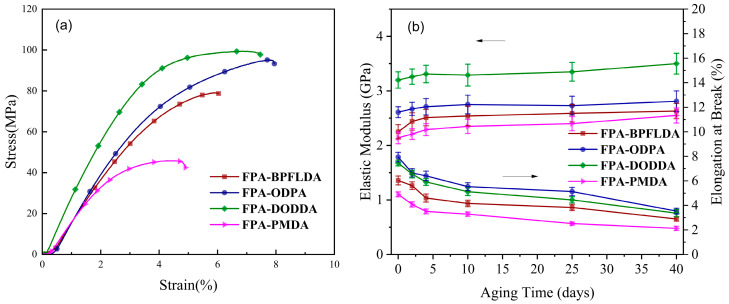
(**a**) Stress–strain curves of unaged FPA-PI films; (**b**) elastic modulus (E_M_, left arrows) and elongation at break (E_B_, right arrows) as a function of aging time for FPA-PI films aged in air at 250 °C for 40 days.

The stress–strain curves of bio-based FPA-PI films are shown in Figure 5a, and the corresponding data are summarized in Table 3. These results demonstrate that all PI films exhibit favorable mechanical properties. The elastic modulus (E_M_) of FPA-PIs ranged from 2.14 to 3.20 GPa, with tensile strength (T_S_) values of 50–99 MPa and elongation at break (E_B_) of 4.9–7.9%. The structure of the dianhydride critically influences mechanical performance. For example, FPA-PMDA showed the poorest mechanical properties, likely due to localized stress imbalance during thermal processing, which induced structural defects in the films. In terms of toughness, FPA-ODPA achieved the highest E_B_ (7.9%), attributable to the flexible ether linkage (-O-) in the ODPA structure [8]. Furthermore, the mechanical properties of these FPA-PI films are comparable to or even surpass those of vanillin-based and citric acid-based bio-polyimides as well as Torlon (poly(amide-imide)) materials reported in the literature [10,54,55], providing crucial theoretical support for developing furan-based bio-polyimides.

### 3.8. Evolution of Elastic Modulus and Elongation at Break in FPA-PI Films with Aging Time

Traditional methods for evaluating the actual service life of polymeric materials, such as natural aging, typically require thousands of hours. Elevated temperature and pressure environments can effectively accelerate oxidative aging processes, thereby significantly reducing testing duration [56]. In accordance with methodologies established by Ruggles-Wrenn and Crochon et al. [57,58], isothermal aging experiments were conducted on FPA-PI films in air at 250 °C for 40 days to investigate the evolution of elastic modulus and elongation at break with aging time (2, 4, 10, 25, and 40 days). As illustrated in Figure 5b, the elastic modulus of FPA-PI films demonstrated an overall increasing trend with prolonged aging duration, while the elongation at break decreased correspondingly. A slow evolution stage was observed between 10 and 25 days for both parameters. Notably, rapid property changes occurred during the initial 0–4 day period, with the elastic modulus increasing by 3.4–8.9% and elongation at break decreasing sharply by 19.3–28%. This accelerated transformation phase can be attributed to interchain crosslinking within the PI molecular structure [55]. Between 25 and 40 days, the films exhibited pronounced embrittlement accompanied by a marked reduction in elongation at break (7.8–20.0%), likely resulting from oxidative degradation and crack formation during prolonged thermal aging.

These findings suggest that FPA-PI films undergo complex structural modifications during high-temperature oxidative aging. It should be noted that the current experiments were conducted below the glass transition temperature (T_g_ = 313 °C). Subsequent investigations could explore aging experiments conducted at temperatures above or below T_g_ to further elucidate the degradation mechanisms of FPA-PI films.

### 3.9. CO_2_/N_2_ Gas Permeability Properties of FPA-PI Films

**Figure 6:**
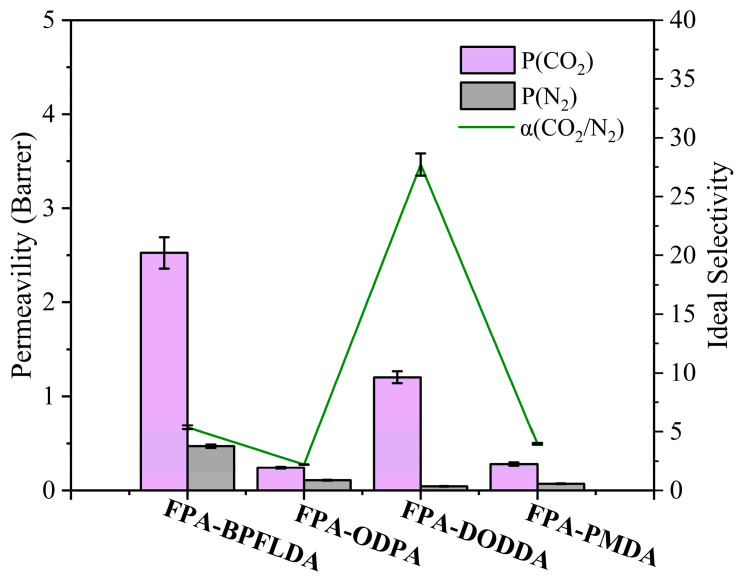
CO_2_ permeability coefficients and CO_2_/N_2_ ideal selectivity of FPA-PI films.

The pure CO_2_ and N_2_ permeability coefficients (P(CO_2_) and P(N_2_)) of FPA-PI films were measured using the pressure difference method, and their ideal CO_2_/N_2_ selectivity (α(CO_2_/N_2_)) was calculated, as shown in Figure 6 and Table 4. Among them, FPA-DODDA exhibited the optimal separation performance, with a selectivity (α(CO_2_/N_2_) = 27.721) surpassing commercial films such as Matrimid^®^ (α = 25.6) [59], Polysulfone (α(CO_2_/N_2_) = 22.400) [60], and Polycarbonate (α(CO_2_/N_2_) = 20.761) [61]. Its CO_2_ permeability (P(CO_2_) = 1.204 Barrer) lies between the highly selective material PBI-I (P(CO_2_) = 0.16 Barrer) [62] and the highly permeable material Polysulfone (P(CO_2_) = 5.6 Barrer). Notably, although cracks were observed on the surface of FPA-DODDA under 100,000× magnification, this structural feature did not result in a significant increase in gas permeability. In contrast, FPA-BPFLDA exhibited higher permeability (P(CO_2_) = 2.53 Barrer), attributed to benzene ring steric effects (d-spacing = 4.7 Å) [63], yet its selectivity (α(CO_2_/N_2_) = 5.4) dropped significantly, consistent with free volume theory predictions. Meanwhile, FPA-ODPA and FPA-PMDA displayed ultralow permeability (P(CO_2_) < 0.3 Barrer) owing to dense chain packing (d-spacing = 3.8–4.3 Å). These FPA-PI films demonstrated a synergistic control mechanism combining size-sieving effects and solution-diffusion processes [64]: (1) The rigid linear DODDA unit promoted tight molecular chain packing (d-spacing = 4.4 Å) with reduced free volume, preferentially hindering the permeation of larger N_2_ molecules (kinetic diameter = 3.64 Å). (2) The majority of solid surfaces preferentially adsorb CO_2_ over N_2_ via a physisorption mechanism. This preference arises from CO_2_’s greater polarizability and quadrupole moment [65], which strengthen its interactions with polar or polarizable surface groups (e.g., -CO-O-CO- in polyimide) through dipole-quadrupole effects. Compared to the poly(amide-imide) Torlon^®^ [66], FPA-PI films exhibited enhanced N_2_ solubility (0.043–0.470 Barrer) but reduced CO_2_ solubility (FPA-ODPA: 0.242 Barrer; FPA-PMDA: 0.282 Barrer), attributed to the denser structure induced by furan ring π-π stacking. The synergy of these mechanisms enables FPA-DODDA to achieve CO_2_/N_2_ selectivity (α = 27.721) comparable to petroleum-based P84 (α = 27.4) [67] while maintaining similar CO_2_ permeability. However, none of these membranes achieved CO2/N2 separation performance reaching the 2008 Robeson upper bound [68].

These results highlight that multi-scale structural modulation of furan rings in bio-based polyimides can overcome traditional film performance limitations, offering a novel strategy for sustainable separation films balancing high selectivity and practical permeability. Notably, Wen et al. [69] confirmed that the incorporation of amide bonds in PIs diminishes gas permeability, which thus may represent another critical factor influencing the CO_2_/N_2_ permeability of the FPA-Pl films in this study. Future studies may adopt design modifications inspired by Torlon^®^ (α(CO_2_/N_2_) = 33.571) to optimize gas permeation flux without compromising selectivity.

## 4. Conclusions

In conclusion, the successful synthesis of the bio-based diamine FPA from renewable FDCA, followed by its polymerization with various dianhydrides, has led to the development of high-performance bio-based polyimide films. These FPA-PI films have demonstrated exceptional thermal stability, with 5% weight loss temperatures (T_d_^5%^) exceeding 425 °C, 10% weight loss temperatures (T_d_^10%^) surpassing 440 °C, and char yields above 54.8%. Mechanically, the films exhibited elastic moduli (2.14–3.20 GPa) and tensile strengths (50–99 MPa) comparable to conventional petroleum-based PIs, alongside robust aging resistance. Notably, FPA-DODDA achieved a CO_2_/N_2_ selectivity of 27.721, outperforming commercial films such as Matrimid^®^, polysulfone, and polycarbonate, while FPA-BPFLDA showed enhanced CO_2_ permeability (P(CO_2_) = 2.526 Barrer), surpassing that of Torlon^®^. The gas separation performance of these films was governed by a synergistic interplay of size-sieving effects and solution-diffusion mechanisms. Despite these advancements, limitations persist: (1) Certain formulations (e.g., FPA-BPAFDA) displayed low reactivity, resulting in film-forming challenges, while the poor solubility of FPA-PIs further restricted processability. (2) The bio-based carbon content of FPA-PIs remained moderate, with a maximum of 21.4% (FPA-PMDA). (3) The dense chain packing (d-spacing = 3.8–4.7 Å) limited CO_2_ permeability, preventing the CO_2_/N_2_ separation performance from reaching the 2008 Robeson upper bound. To address these limitations, future efforts should focus on developing fully bio-based systems, incorporating flexible chains, and introducing bulky substituents to optimize free volume and processability. Overall, this study validates the potential of bio-based polyimides as environmentally friendly and sustainable alternatives to traditional petroleum-derived polyimides, offering valuable insights for the future design and application of green, high-performance materials.

## Figures and Tables

**Table 1:** Intrinsic viscosities ([η]) ^a^ of FPA-PAAs; rough simulation and calculation of densities (ρ) ^b^ and solubilities ^c^ of FPA-PIs.

FPA-PIs	[η] (dL·g^−1^)	ρ [g·cm^−3^]	NMP	DMF	DMAC	DMSO	DCM	CHCl_3_	THF	Acetone
FPA-BPFLDA	2.38 ^d^	1.298	--	--	--	--	--	--	--	--
FPA-DODDA	0.60 ^d^	1.341	--	--	--	--	--	--	--	--
FPA-PMDA	1.08 ^d^	1.390	--	--	--	--	--	--	--	--
FPA-ODPA	0.93 ^d^	1.391	--	--	--	--	--	--	--	--
FPA-SDPDA	0.28 ^e^	1.388	X	X	X	X	X	X	X	X
FPA-BPAFDA	0.26 ^e^	1.420	X	X	X	X	X	X	X	X
FPA-BTDA	0.49 ^e^	1.393	X	X	X	X	X	X	X	X
FPA-HQDA	1.23 ^e^	1.366	X	X	X	X	X	X	X	X

^a^ Intrinsic viscosity ([η]) measurement: the intrinsic viscosities of FPA-PAAs were measured using an Ubbelohde viscometer with N-methyl-2-pyrrolidone (NMP) as the solvent at 25.0 °C ± 0.1 °C and a PAA concentration of 0.5 g·dL^−1^. ^b^ Density of FPA-PI films rapidly predicted using the Synthia module in Materials Studio was overestimated compared to experimental values. ^c^ Solubility testing: each polyimide film (10 mg) was immersed in 1 mL of solvent (NMP, DMF, DMAC, DMSO, DCM, chloroform (CHCl_3_), THF, or acetone) at room temperature and stirred for 24 h. Solubility tests showed the films were insoluble (‘--’); untested ones are marked ‘X’. ^d^ Film formation (successful): films were successfully prepared via thermal imidization. ^e^ Film formation (failed): films failed to form through thermal imidization.

**Table 2:** Thermal properties from DSC and TGA of FPA-PI films.

FPA-PIs	Bio-C (%) ^a^	T_g_ (°C) ^b^	T_d_^5%^ (°C) ^c^	T_d_^10%^ (°C) ^d^	Char Yield (%) ^e^
FPA-BPFLDA	10.2	313	438	463	57.8
FPA-ODPA	17.6	N.F.	463	484	59.6
FPA-DODDA	13.0	N.F.	425	440	55.7
FPA-PMDA	21.4	N.F.	475	504	54.8

^a^ Bio-based content (Bio-C) in the polyimide. ^b^ Glass transition temperature (T_g_); N.F.: not found. ^c^ Temperature at 5% weight loss (T_d_^5%^). ^d^ Temperature at 10% weight loss (T_d_^10%^). ^e^ Char yield: carbon residue remaining after pyrolysis.

**Table 3:** Mechanical properties of FPA-PI films.

FPA-PIs	E_M_ (GPa) ^a^	T_S_ (MPa) ^b^	E_B_ (%) ^c^
FPA-BPFLDA	2.25 ± 0.13	79.03 ± 4.19	6.03 ± 0.36
FPA-ODPA	2.61 ± 0.10	95.22 ± 4.11	7.94 ± 0.38
FPA-DODDA	3.20 ± 0.15	99.43 ± 4.06	7.46 ± 0.23
FPA-PMDA	2.14 ± 0.11	50.47 ± 1.80	4.90 ± 0.20

^a^ E_M_: Elastic modulus; ^b^ T_S_: tensile strength; ^c^ E_B_: elongation at break.

**Table 4:** CO_2_ permeability coefficients and CO_2_/N_2_ ideal selectivity data of FPA-PI films.

FPA-PIs	P(CO_2_) (Barrer) ^a^	P(N_2_) (Barrer) ^b^	α(CO_2_/N_2_) ^c^
FPA-BPFLDA	2.526 ± 0.167	0.470 ± 0.017	5.368 ± 0.167
FPA-ODPA	0.242 ± 0.010	0.109 ± 0.004	2.215 ± 0.018
FPA-DODDA	1.204 ± 0.065	0.043 ± 0.003	27.721 ± 0.953
FPA-PMDA	0.282 ± 0.017	0.071 ± 0.004	3.963 ± 0.073
Matrimid^®^ 5218 [59]	6.5	0.25	25.6
Polysulfone [60]	5.6	0.25	22.400 ^d^
Polycarbonate [61]	6.0	0.289	20.761 ^d^
PBI-I [62]	0.16	0.0048	33.333 ^d^
Torlon^®^ [66]	0.47	0.014	33.571 ^d^
P84 [67]	1.37	0.050	27.400 ^d^

^a^ CO_2_ permeability coefficient (P(CO_2_)). ^b^ N_2_ permeability coefficient (P(N_2_)), where 1 Barrer = 1 × 10^−10^ cm^3^ (STP)·cm·cm^−2^·s^−1^·cmHg^−1^. ^c^ Ideal selectivity (α(CO_2_/N_2_)) for CO_2_/N_2_ separation by the film. ^d^ Calculated from data reported in the literature, rounded to three decimal places.

## Data Availability

The data presented in this study are available on request from the corresponding author.

## Supplementary Materials

The following supporting information can be downloaded at https://www.mdpi.com/article/10.3390/polym17101362/s1, ball-and-stick models of the repeating unit of FPA-Pls (Figure S1); NMR spectra of bio-based monomer precursors, bio-based monomer (FPA), dianhydride precursors, and dianhydrides (Figures S2 to S29).

## References

[1] Liu L., Duan Y., Yun H., Chen X., Liu J., Lv S., Zhang Y. (2024). Progress on the research and development of the biomass-based polyimide. Ind. Crops Prod..

[2] Chen Y., Fan S., Yi X., Li B., Chen S., Liu S., Hu T., Chen S. (2021). Preparation and Property of Bio-Polyimide/Halloysite Nanocomposite Based on 2,5-Furandicarboxylic Acid. Polymers.

[3] Marshall A., Jiang B., Gauvin R.M., Thomas C.M. (2022). 2,5-Furandicarboxylic Acid: An Intriguing Precursor for Monomer and Polymer Synthesis. Molecules.

[4] Kashparova V.P., Chernysheva D.V., Klushin V.A., Andreeva V.E., Kravchenko O.A., Smirnova N.V. (2021). Furan monomers and polymers from renewable plant biomass. Russ. Chem. Rev..

[5] Su Y.-K., Shorta G.N., Miller S.A. (2023). Renewable and water-degradable polyimide-esters from citric acid. Green Chem..

[6] Sava I., Damaceanu M.-D., Constantin C.-P., Asandulesa M., Wolińska-Grabczyk A., Jankowski A. (2018). Structure–promoted high performance properties of triphenylmethane-containing polyimides and copolyimides. Eur. Polym. J..

[7] Liu X.-J., Zheng M.-S., Chen G., Dang Z.-M., Zha J.-W. (2022). High-temperature polyimide dielectric materials for energy storage: Theory, design, preparation and properties. Energy Environ. Sci..

[8] Yang Z., Zhang Y., Li S., Zhang X., Wang T., Wang Q. (2020). Fully Closed-Loop Recyclable Thermosetting Shape Memory Polyimide. ACS Sustain. Chem. Eng..

[9] Mai A.T.M., Thakur A., Ton N.N.T., Nguyen T.N., Kaneko T., Taniike T. (2021). Photodegradation of a semi-aromatic bio-derived polyimide. Polym. Degrad. Stab..

[10] Jiang X., Long Y., Chen K., Yu Q., Jiang L., Chi Z., Liu S., Xu J., Zhang Y. (2023). Preparation, characterization, and bio-degradation studies of high-performance bio-based polyimides based on bicyclic diamines derived from citric acid. J. Mater. Chem. C.

[11] Lin H., Fan H., Yang C., Zhu S., Xie T., Xiang C., Yao H., Guan S. (2025). Porous polyimide films with low dielectric constant prepared by integrated strategy containing construction of pore structure and crosslinking network engineering. Polymer.

[12] Wang Q., Li X., Kan M., Gao H., Liu S., Ji X., Mu H., Mao Z., Yuan Z. (2025). Lignin enhanced shape memory polyimide with superior mechanical property and performance. Chem. Eng. J..

[13] Tsurusaki Y., Sawada R., Liu H., Ando S. (2025). Optical, Dielectric, and Thermal Properties of Bio-Based Polyimides Derived from An Isosorbide-Containing Diamine. Macromol. Rapid Commun..

[14] Zhang Y., Chen L., He Y., Luo W., Li K., Min Y. (2023). Synthesis of Furan-Based Diamine and Its Application in the Preparation of Bio-Based Polyimide. Polymers.

[15] Troiano D., Orsat V., Dumont M.-J. (2020). Status of Biocatalysis in the Production of 2,5-Furandicarboxylic Acid. ACS Catal..

[16] Ma K., Chen G., Wang W., Zhang A., Zhong Y., Zhang Y., Fang X. (2018). Partially bio-based aromatic polyimides derived from 2,5-furandicarboxylic acid with high thermal and mechanical properties. J. Polym. Sci. A Polym. Chem..

[17] Favvas E.P., Katsaros F.K., Papageorgiou S.K., Sapalidis A.A., Mitropoulos A.C. (2017). A review of the latest development of polyimide based membranes for CO_2_ separations. React. Funct. Polym..

[18] Hu X., Lee W.H., Zhao J., Bae J.Y., Kim J.S., Wang Z., Yan J., Zhuang Y., Lee Y.M. (2020). Tröger’s Base (TB)-containing polyimide membranes derived from bio-based dianhydrides for gas separations. J. Membr. Sci..

[19] Chen G., Li D., Chen L., Lin Z., Li W., Zhao B., Zhao Z., Liu J., Sun Y., Pang J. (2024). Innovative thermal crosslinked polyimide gas separation membrane with highly selective and resistance to physical aging base on phenyl ethynyl. Chem. Eng. J..

[20] Sanaeepur H., Amooghin A.E., Bandehali S., Moghadassi A., Matsuura T., Van der Bruggen B. (2019). Polyimides in membrane gas separation: Monomer’s molecular design and structural engineering. Prog. Polym. Sci..

[21] Huang Y., Li K., Zhang Y., Wang G., Ma Y., Jiao L., Shu D., Yang S., Ma X., Zhang Q. (2025). Fine-tuning gas separation performance of copolymer polyimide by the regulation of local microstructure. J. Membr. Sci..

[22] Bei P., Liu H., Zhang Y., Gao Y., Cai Z., Chen Y. (2021). Preparation and characterization of polyimide membranes modified by a task-specific ionic liquid based on Schiff base for CO_2_/N_2_ separation. Environ. Sci. Pollut. Res..

[23] Yan J., Zhang B., Wang Z. (2018). Highly Selective Separation of CO_2_, CH_4_, and C_2_–C_4_ Hydrocarbons in Ultramicroporous Semicycloaliphatic Polyimides. ACS Appl. Mater. Interfaces.

[24] Chuah C.Y., Lee J., Song J., Bae T.-H. (2020). CO_2_/N_2_ Separation Properties of Polyimide-Based Mixed-Matrix Membranes Comprising UiO-66 with Various Functionalities. Membranes.

[25] Suvannasara P., Tateyama S., Miyasato A., Matsumura K., Shimoda T., Ito T., Yamagata Y., Fujita T., Takaya N., Kaneko T. (2014). Biobased Polyimides from 4-Aminocinnamic Acid Photodimer. Macromolecules.

[26] Michael A.J. (2016). Biosynthesis of polyamines and polyamine-containing molecules. Biochem. J..

[27] Hsiao S.H., Chung C.L., Lee M.L. (2004). Synthesis and characterization of soluble polyimides derived from 2′,5′-bis(3,4-dicarboxyphenoxy)-p-terphenyl dianhydride. Polym. Sci. A Polym. Chem..

[28] Liaw D.-J., Liaw B.-Y., Hsu P.-N., Hwang C.-Y. (2001). Synthesis and Characterization of New Highly Organosoluble Poly(ether imide)s Bearing a Noncoplanar 2,2′-Dimethyl-4,4′-biphenyl Unit and Kink Diphenylmethylene Linkage. Chem. Mater..

[29] Liu C., Wang J., Lin E., Zong L., Jian X. (2012). Synthesis and properties of phthalonitrile-terminated oligomeric poly(ether imide)s containing phthalazinone moiety. Polym. Degrad. Stab..

[30] Terraza C.A., Ortiz P., Tagle L.H., Pérez G., Saldias C., Rodríguez-González F.E., Cabrera-Barjas G., Catalán H., Tun-didor-Camba A., Coll D. (2019). Synthesis and Properties of Poly(imides) and Poly(imides)/Ionic Liquid Composites Bearing a Ben-zimidazole Moiety. Polymers.

[31] Bao F., Qiu L., Zou B., Lei H., Peng W., Cheng S.Z.D., Huang M. (2024). Development of Fluorinated Colorless Polyimides of Restricted Dihedral Rotation toward Flexible Substrates with Thermal Robustness. Macromolecules.

[32] (2017). Standard Test Method for Oxygen Gas Transmission Rate Through Plastic Film and Sheeting Using a Coulometric Sensor.

[33] Ohya S., Fujii Y., Yao S. (2007). Creation of porous polyimide membrane by viscoelastic phase separation. Nihon Reoroji Gakk..

[34] Zhang W., Wu Q., Shao W., Li F., Chen H., Pei Y., Wang J. (2023). Soluble and cross-linkable polyimides from a vanillin-derived diamine: Preparation, post-polymerization and properties. Polym. Chem..

[35] Lee J., Baek S., Kim J., Lee S., Kim J., Han H. (2021). Highly Soluble Fluorinated Polyimides Synthesized with Hydrothermal Process towards Sustainable Green Technology. Polymers.

[36] Shin S.-R., Moon S.-Y., Park C.-Y., Chang B.-J., Kim J.-H. (2018). Solution-processable methyl-substituted semi-alicyclic homo- and co-polyimides and their gas permeation properties. Polymer.

[37] Soh L.S., Hong S.U., Liang C.Z., Yong W.F. (2023). Green solvent-synthesized polyimide membranes for gas separation: Coupling Hansen solubility parameters and synthesis optimization. Chem. Eng. J..

[38] Otárola-Sepúlveda J., Cea-Klapp E., Aravena P., Ormazábal-Latorre S., Canales R.I., Garrido J.M., Valerio O. (2023). Assessment of Hansen solubility parameters in deep eutectic solvents for solubility predictions. J. Mol. Liq..

[39] Zhang P., Zhao J., Zhang K., Wu Y., Li Y. (2018). Effect of co-solvent on the structure and dielectric properties of porous polyimide membranes. J. Phys. D Appl. Phys..

[40] Liao R., Guo Y., Yang L., Zhou H., Jin W. (2023). Solvent-induced microstructure of polyimide membrane to enhance CO_2_/CH_4_ separation. J. Membr. Sci..

[41] Wu Y., Ji J., Huang H., Liu S., Zhao J. (2021). Facile synthesis of acyloxy-containing fluorene-based Cardo polyimides with high optical transparency, fluorescence and low dielectric constant. React. Funct. Polym..

[42] Zhang Q., Song H., Gao C.-F. (2024). The intrinsic relationship of the thermal stress intensity factor and the temperature difference at the crack surface. J. Therm. Stress..

[43] Zhu G., Lao H., Feng F., Wang M., Fang X., Chen G. (2022). Synthesis and characterization of poly(amide-imide)s with high T_g_ and low CTE derived from isomeric amide-containing diamines. Eur. Polym. J..

[44] Chen C.-K., Lin Y.-C., Miyane S., Ando S., Ueda M., Chen W.-C. (2020). Thermally and Mechanically Stable Polyimides as Flexible Substrates for Organic Field-Effect Transistors. ACS Appl. Polym. Mater..

[45] Ma X., Swaidan R., Belmabkhout Y., Zhu Y., Litwiller E., Jouiad M., Pinnau I., Han Y. (2012). Synthesis and Gas Transport Properties of Hydroxyl-Functionalized Polyimides with Intrinsic Microporosity. Macromolecules.

[46] Wang Y., Lu Y., Zhang J., Liang Y., Chi H., Xiao G. (2021). Enhanced toughness and gas permeabilities of polyimide composites derived from polyimide matrix and flower-like polyimide microparticles. Polym. Compos..

[47] Siracusa C., Quartinello F., Soccio M., Manfroni M., Lotti N., Dorigato A., Guebitz G.M., Pellis A. (2023). On the Selective Enzymatic Recycling of Poly(pentamethylene 2,5-furanoate)/Poly(lactic acid) Blends and Multiblock Copolymers. ACS Sustain. Chem. Eng..

[48] Bicerano J. (2002). Prediction of Polymer Properties.

[49] Park C.-Y., Kim E.-H., Kim J.H., Lee Y.M., Kim J.-H. (2018). Novel semi-alicyclic polyimide membranes: Synthesis, characterization, and gas separation properties. Polymer.

[50] Wang Y.-H., Hung D.-Y., Liu Y.-L. (2024). Is a Vitrimer with a High Glass Transition Temperature Available? A Case Study on Rigid Polyimides Cross-Linked with Dynamic Ester Bonds. Macromol. Rapid Commun..

[51] Krishnan P.S.G., Veeramani S. (2003). Effect of methyl group substitution in the diamine and copolymer composition on thermal degradation of copolyimides based on 2,2-bis(3,4-dicarboxyphenyl) hexafluoropropane dianhydride. Polym. Degrad. Stab..

[52] Yerzhankyzy A., Wang Y., Ghanem B.S., Puspasari T., Pinnau I. (2022). Gas separation performance of solid-state in-situ thermally crosslinked 6FDA-based polyimides. J. Membr. Sci..

[53] Norton G.A., Devlin S.L. (2006). Determining the modern carbon content of biobased products using radiocarbon analysis. Bioresour. Technol..

[54] Wang Z., Li Y., Zhu T., Xiong L., Liu F., Qi H. (2019). Conversion of renewable vanillin into high performance polyimides via an asymmetric aromatic diamine derivation. Polym. Degrad. Stab..

[55] Tang A., Chen Z., Nie H., Dong J., Zhao X., Li X., Xu Q., Zhang Q. (2025). Polyimide gas separation membrane with an ultrahigh molecule sieving ability by interchain hydrogen-bonding and thermo-oxidative cross-linking networks. Sep. Purif. Technol..

[56] An N., Pochiraju K.V., Tandon G.P., Proulx T. (2011). Accelerated Testing Methods for Oxidative Aging of Polymeric Composites. Mechanics of Time-Dependent Materials and Processes in Conventional and Multifunctional Materials.

[57] Ruggles-Wrenn M.B., Broeckert J.L. (2009). Effects of Prior Aging at 2888C in Air and in Argon Environments on Creep Response of PMR-15 Neat Resin. J. Appl. Polym. Sci..

[58] Crochon T., Li C., Lévesque M. (2015). Ontime-temperature-dependent viscoelastic behavior of an amorphous polyimide. Mech. Time-Depend. Mater..

[59] Tin P.S., Chung T.S., Liu Y., Wang R., Liu S.L., Pramoda K.P. (2003). Effects of cross-linking modification on gas separation performance of Matrimid membranes. J. Membr. Sci..

[60] McHattie J.S., Koros W.J., Paul D.R. (1991). Gas transport properties of polysulphones: 1. Role of symmetry of methyl group placement on bisphenol rings. Polymer.

[61] Muruganandam N., Koros W.J., Paul D.R. (1987). Gas Sorption and Transport in Substituted Polycarbonates. J. Polym. Sci. Pol. Phys..

[62] Kumbharkar S.C., Karadkar P.B., Kharul U.K. (2006). Enhancement of gas permeation properties of polybenzimidazoles by systematic structure architecture. J. Membr. Sci..

[63] Sawada R., Ando S. (2022). Colorless, Low Dielectric, and Optically Active Semialicyclic Polyimides Incorporating a Biobased Isosorbide Moiety in the Main Chain. Macromolecules.

[64] Pandey P., Chauhan R.S. (2001). Membranes for gas separation. Prog. Polym. Sci..

[65] McDonald T.M., D’Alessandro D.M., Krishna R., Long J.R. (2011). Enhanced carbon dioxide capture upon incorporation of N,N′-dimethylethylenediamine in the metal-organic framework CuBTTri. Chem. Sci..

[66] Kosuri M.R., Koros W.J. (2008). Defect-free asymmetric hollow fiber membranes from Torlon^®^, a polyamide–imide polymer, for high-pressure CO_2_ separations. J. Membr. Sci..

[67] Hosseini S.S., Chung T.S. (2009). Carbon membranes from blends of PBI and polyimides for N_2_/CH_4_ and CO_2_/CH_4_ separation and hydrogen purification. J. Membr. Sci..

[68] Robeson L.M. (2008). The upper bound revisited. J. Membr. Sci..

[69] Wen Q., Tang A., Chen C., Liu Y., Xiao C., Tan J., Li D. (2021). Impact of Backbone Amide Substitution at the Meta- and Pa-ra-Positions on the Gas Barrier Properties of Polyimide. Materials.

