# Supplementary material for: Preparation, Thermal Stability, and Preliminary Gas Separation Performance of Furan-Based Bio-Polyimide Films
Source: Polymers (Basel). 2025 May 16;17(10):1362. doi: 10.3390/polym17101362 (PMC12115040; doi:10.3390/polym17101362)
Supplement: Supplementary file 1 [file polymers-17-01362-s001.zip › polymers-3604312-supplementary.pdf]

Preparation, Thermal Stability, and Preliminary Gas Separation Performance of Furan-Based Bio-Polyimide Films

Contents	Pages
**Figure S1. Ball-and-stick models of the repeating unit of FPA-PIs**	2
**Figure S2. ^{1}H NMR spectrum of FDCDCl in CDCl3**	3
**Figure S3. ^{13}C NMR spectrum of FDCDCl in CDCl3**	3
**Figure S4. ^{1}H NMR spectrum of FPN in DMSO-d6**	4
**Figure S5. ^{13}C NMR spectrum of FPN in DMSO-d6**	4
**Figure S6. ^{1}H NMR spectrum of FPA in DMSO-d6**	5
**Figure S7. ^{13}C NMR spectrum of FPA in DMSO-d6**	5
**Figure S8. ^{1}H NMR spectrum of BPFLCN in DMSO-d6**	6
**Figure S9. ^{13}C NMR spectrum of BPFLCN in DMSO-d6**	6
**Figure S10. ^{1}H NMR spectrum of HQCN in DMSO-d6**	7
**Figure S11. ^{13}C NMR spectrum of HQCN in DMSO-d6**	7
**Figure S12. ^{1}H NMR spectrum of SDPCN in DMSO-d6**	8
**Figure S13. ^{13}C NMR spectrum of SDPCN in DMSO-d6**	8
**Figure S14. ^{1}H NMR spectrum of DODCN in DMSO-d6**	9
**Figure S15. ^{13}C NMR spectrum of DODCN in DMSO-d6**	9
**Figure S16. ^{1}H NMR spectrum of BPAFCN in DMSO-d6**	10
**Figure S17. ^{13}C NMR spectrum of BPAFCN in DMSO-d6**	10
**Figure S18. ^{19}F NMR spectrum of BPAFCN in DMSO-d6**	11
**Figure S19. ^{1}H NMR spectrum of BPAFDA in DMSO-d6**	11
**Figure S20. ^{13}C NMR spectrum of BPAFDA in DMSO-d6**	12
**Figure S21. ^{19}F NMR spectrum of BPAFDA in DMSO-d6**	12
**Figure S22. ^{1}H NMR spectrum of BPFLDA in DMSO-d6**	13
**Figure S23. ^{13}C NMR spectrum of BPFLDA in DMSO-d6**	13
**Figure S24. ^{1}H NMR spectrum of HQDA in DMSO-d6**	14
**Figure S25. ^{13}C NMR spectrum of HQDA in DMSO-d6**	14
**Figure S26. ^{1}H NMR spectrum of SDPDA in DMSO-d6**	15
**Figure S27. ^{13}C NMR spectrum of SDPDA in DMSO-d6**	15
**Figure S28. ^{1}H NMR spectrum of DODDA in DMSO-d6**	16
**Figure S29. ^{13}C NMR spectrum of DODDA in DMSO-d6**	16

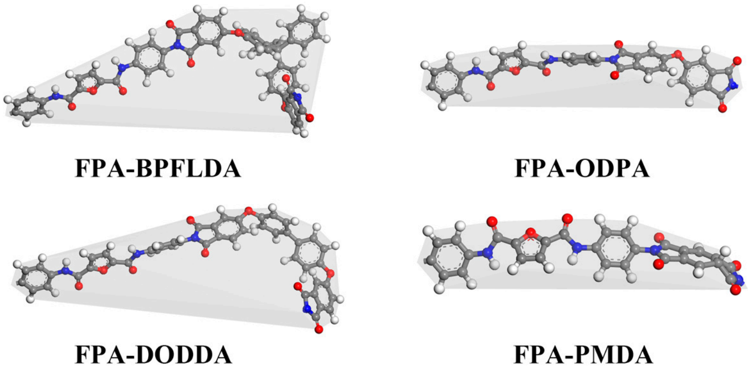

Figure S1. Ball-and-stick models of the repeating unit of FPA-PIs

NMR Spectra of Bio-Based Monomer Precursors, Bio-Based Monomer (FPA), Dianhydride Precursors, and Dianhydrides

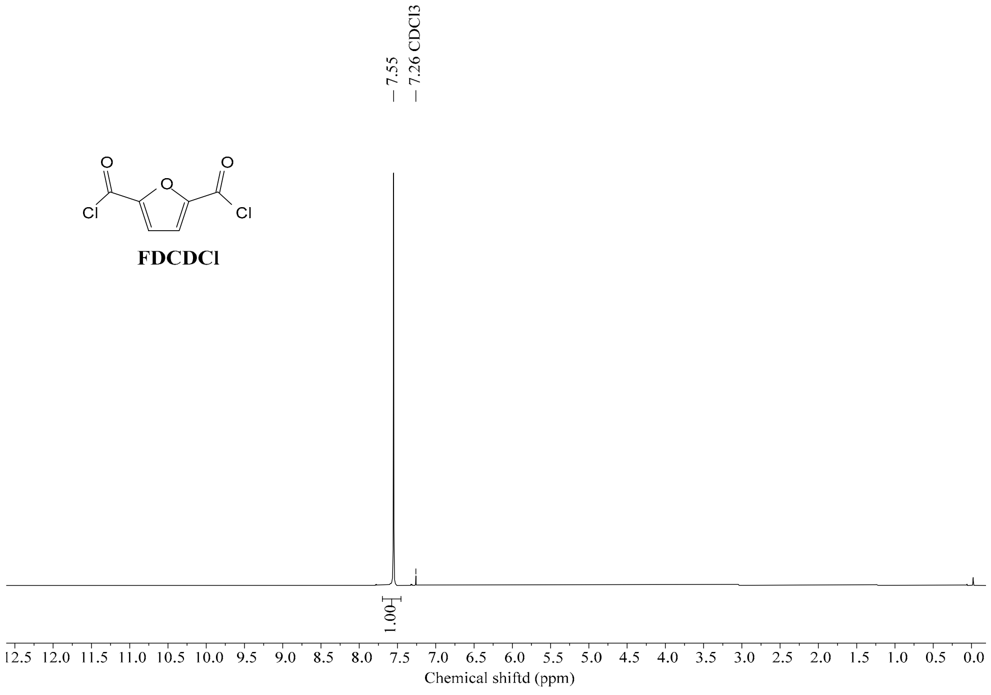

Figure S2. ^{1}H NMR spectrum of FDCDCl in $CDCl_3$

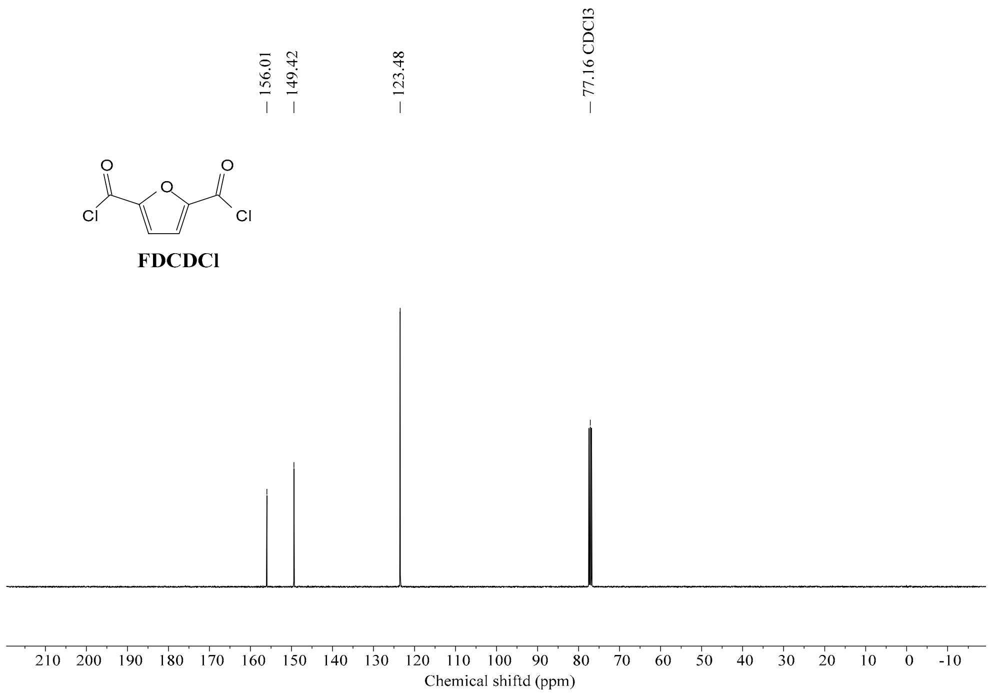

Figure S3. ^{13}C NMR spectrum of FDCDCl in $CDCl_3$

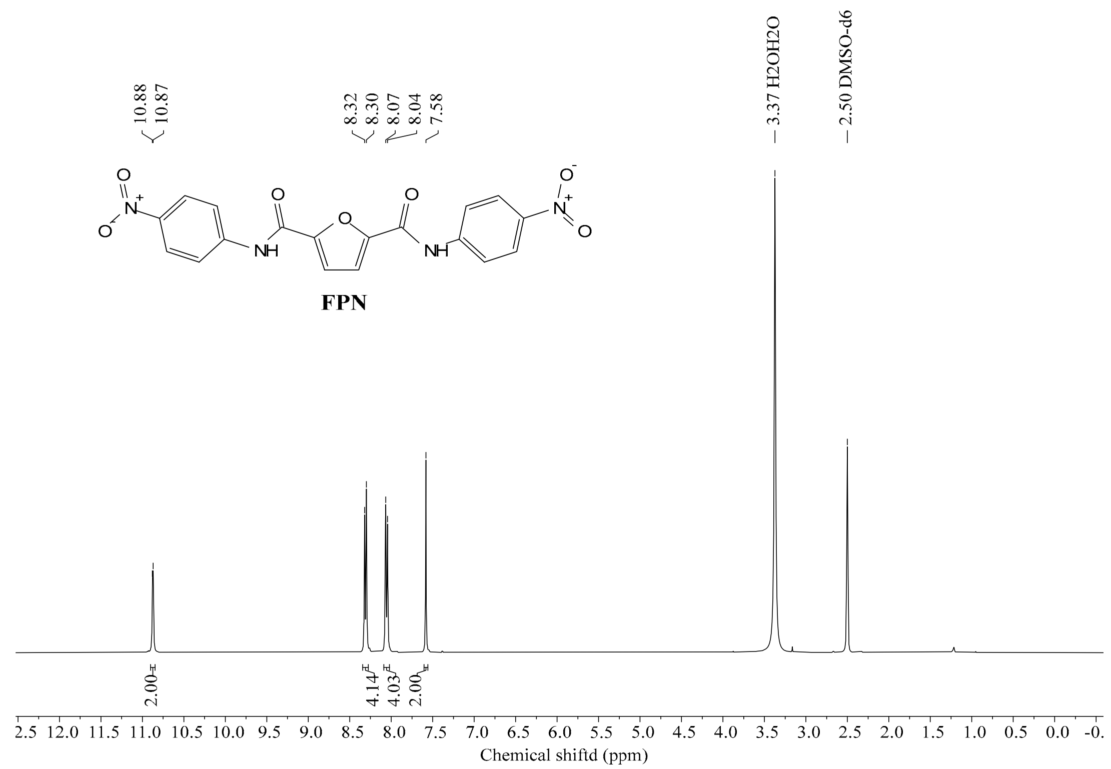

Figure S4. ^{1}H NMR spectrum of FPN in DMSO-*d6*

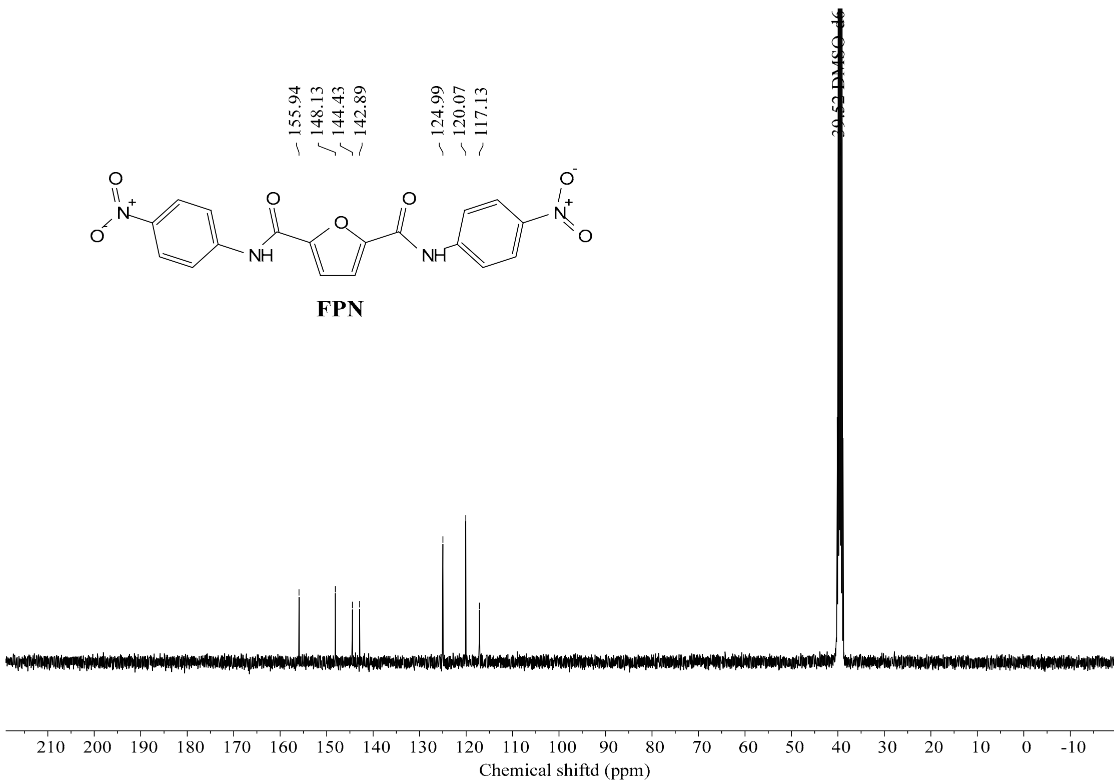

Figure S5. ^{13}C NMR spectrum of FPN in DMSO-*d6*

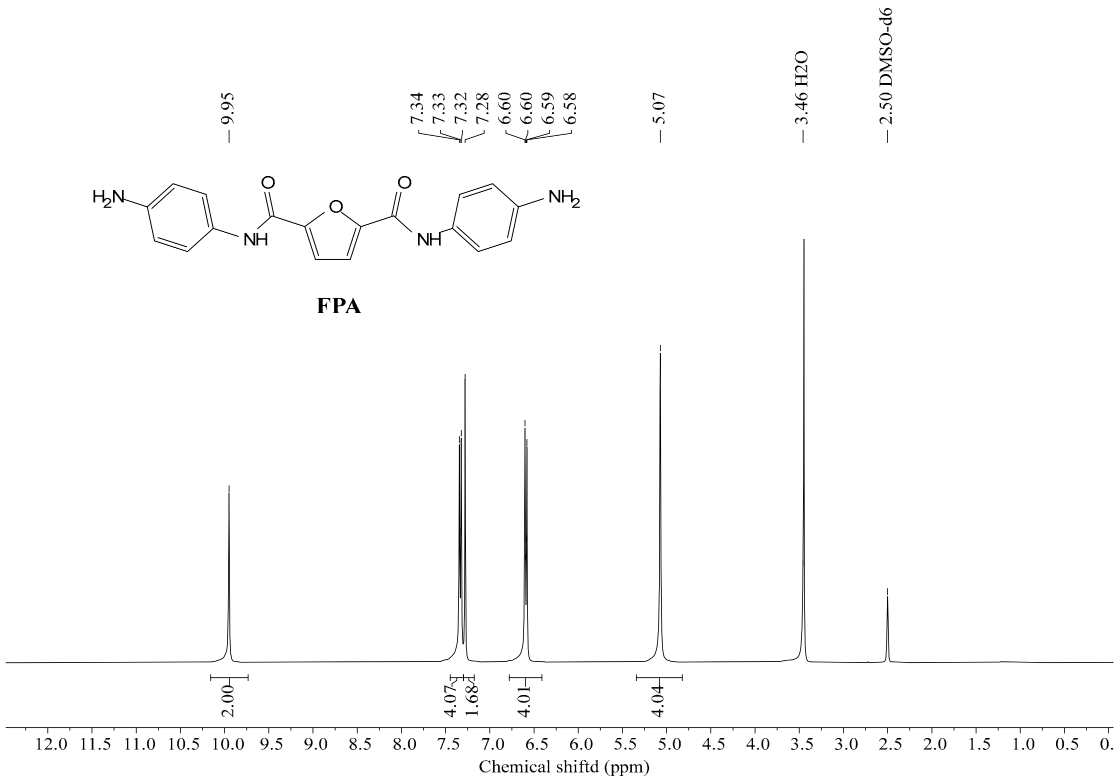

Figure S6. ^{1}H NMR spectrum of FPA in DMSO-*d6*

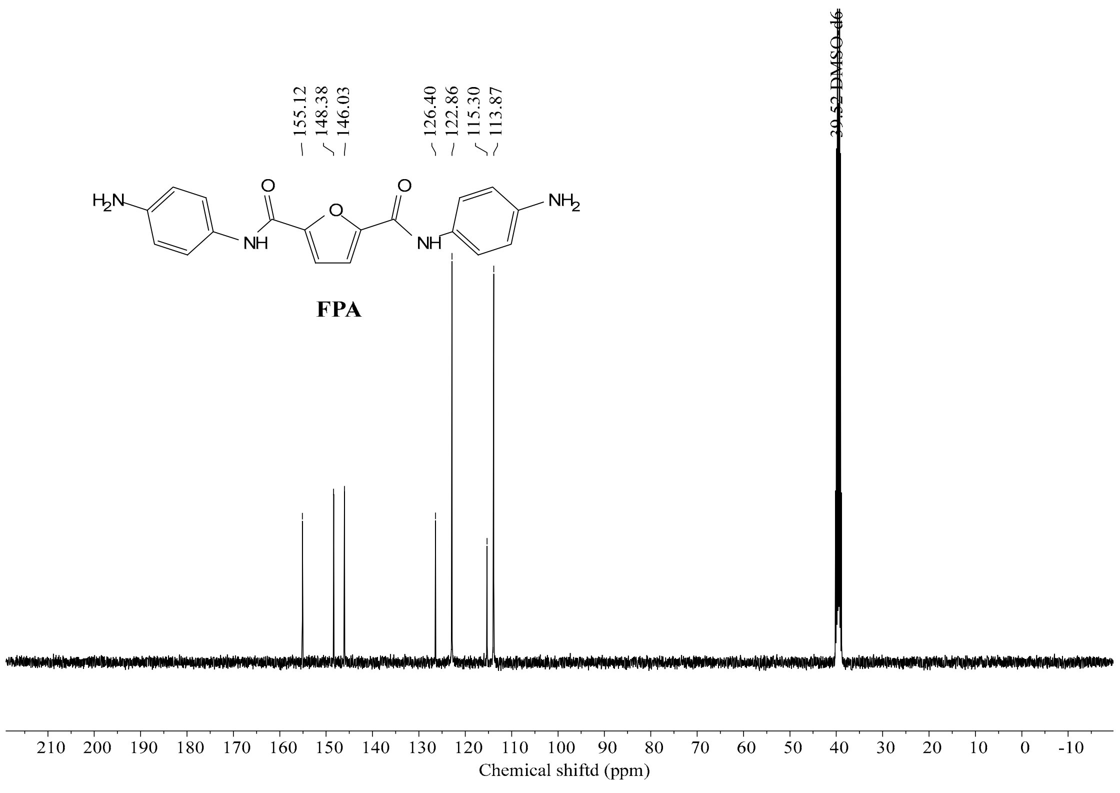

Figure S7. ^{13}C NMR spectrum of FPA in DMSO-*d6*

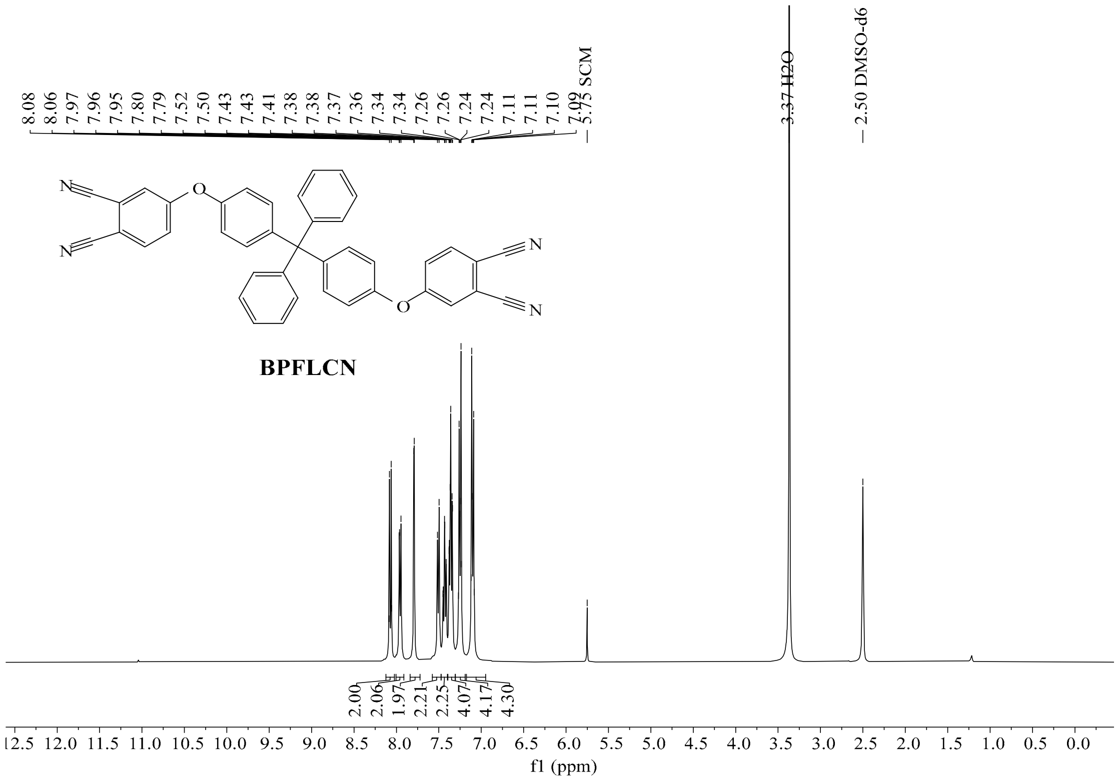

Figure S8. ^{1}H NMR spectrum of BPFLCN in DMSO-*d6*

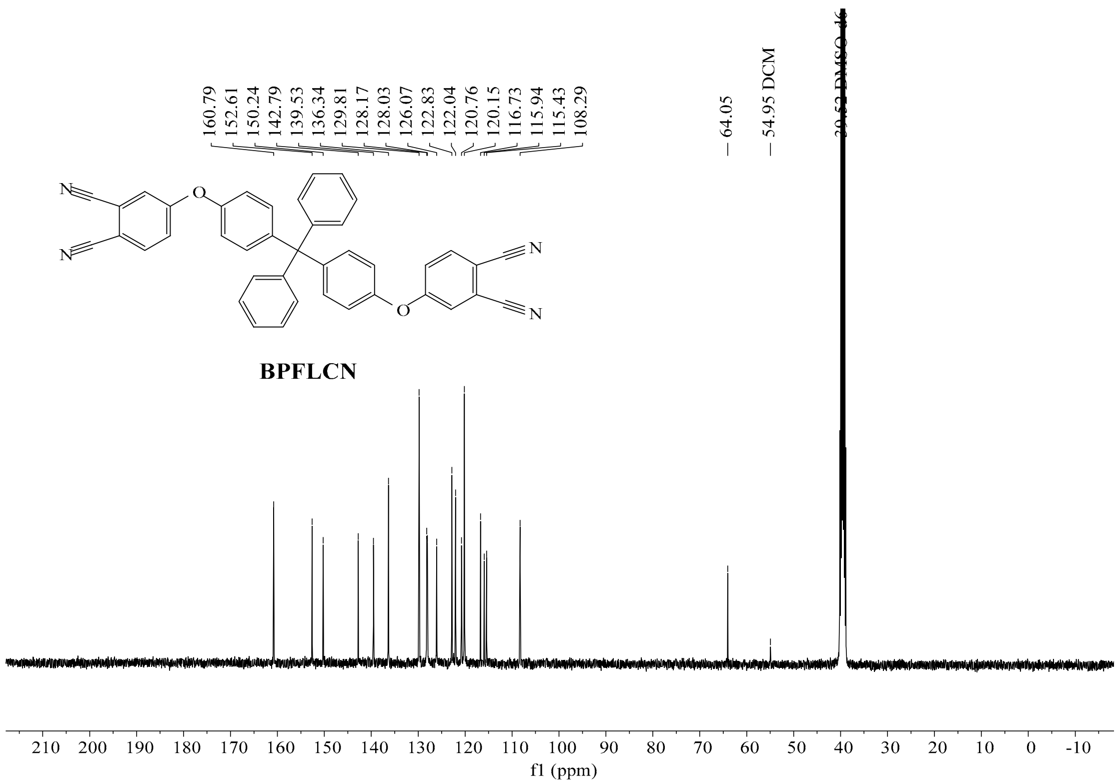

Figure S9. ^{13}C NMR spectrum of BPFLCN in DMSO-*d6*

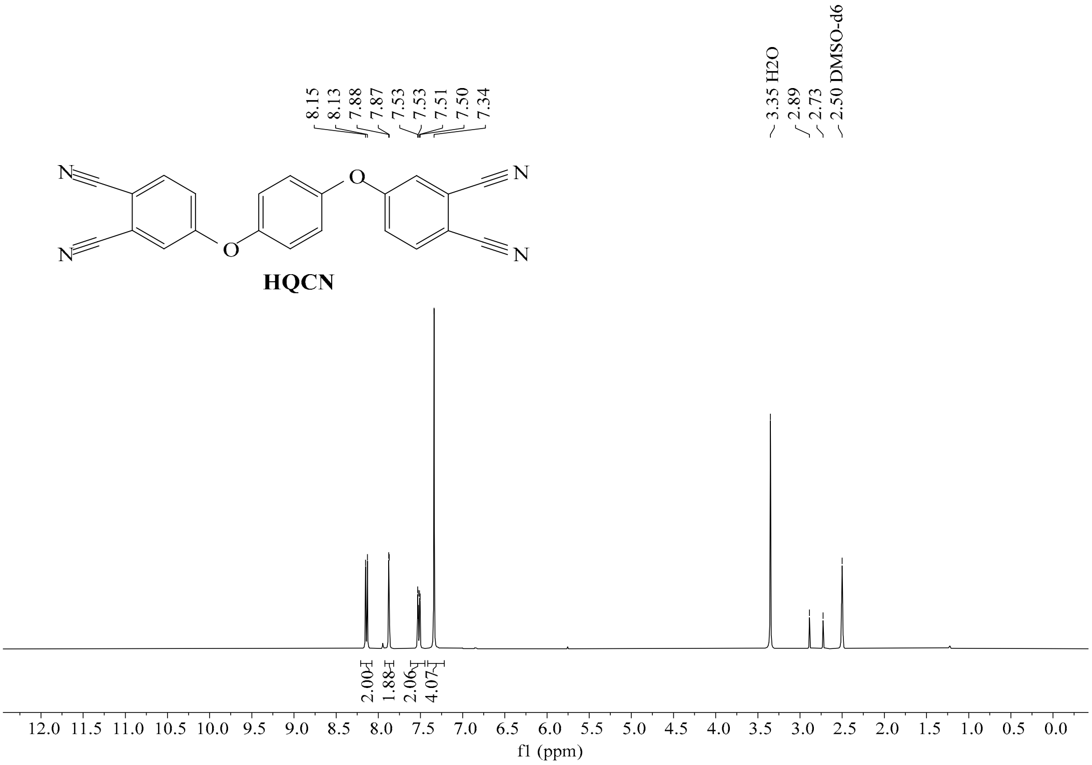

Figure S10. ^{1}H NMR spectrum of HQCN in DMSO-*d6*

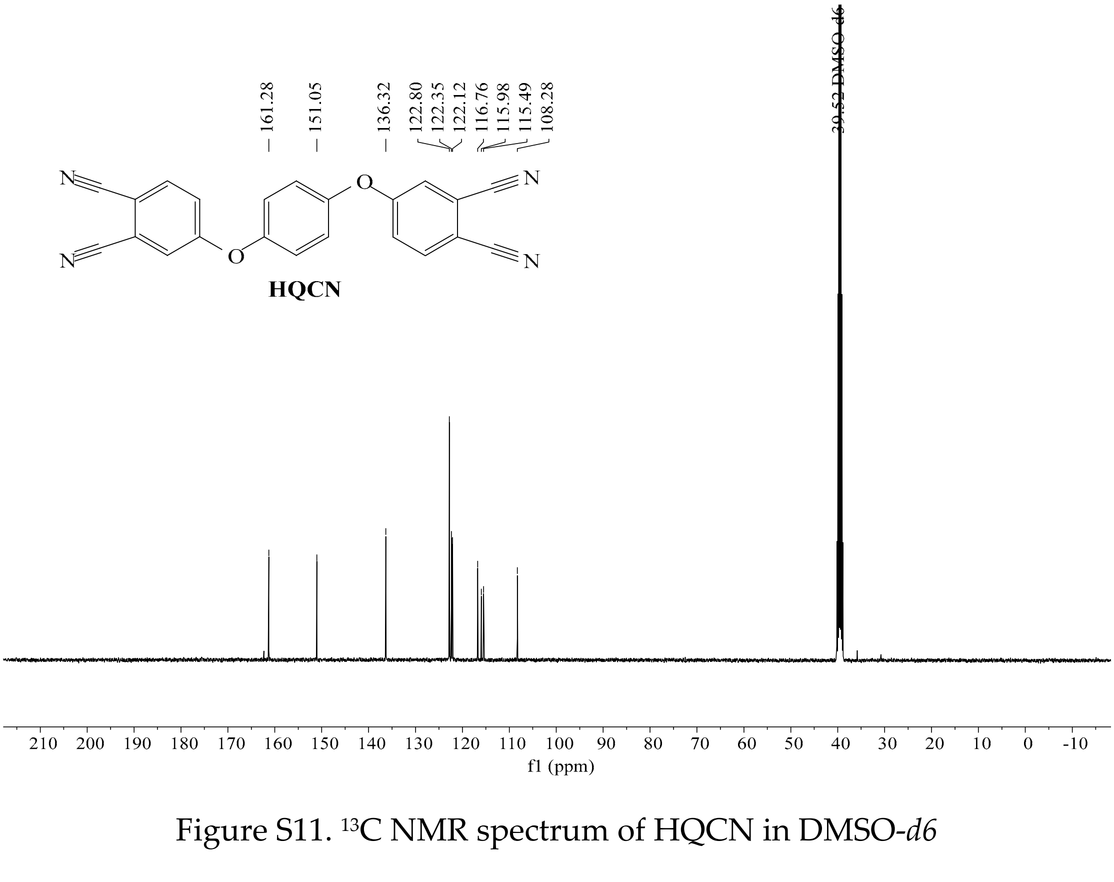

Figure S11. ^{13}C NMR spectrum of HQCN in DMSO-*d6*

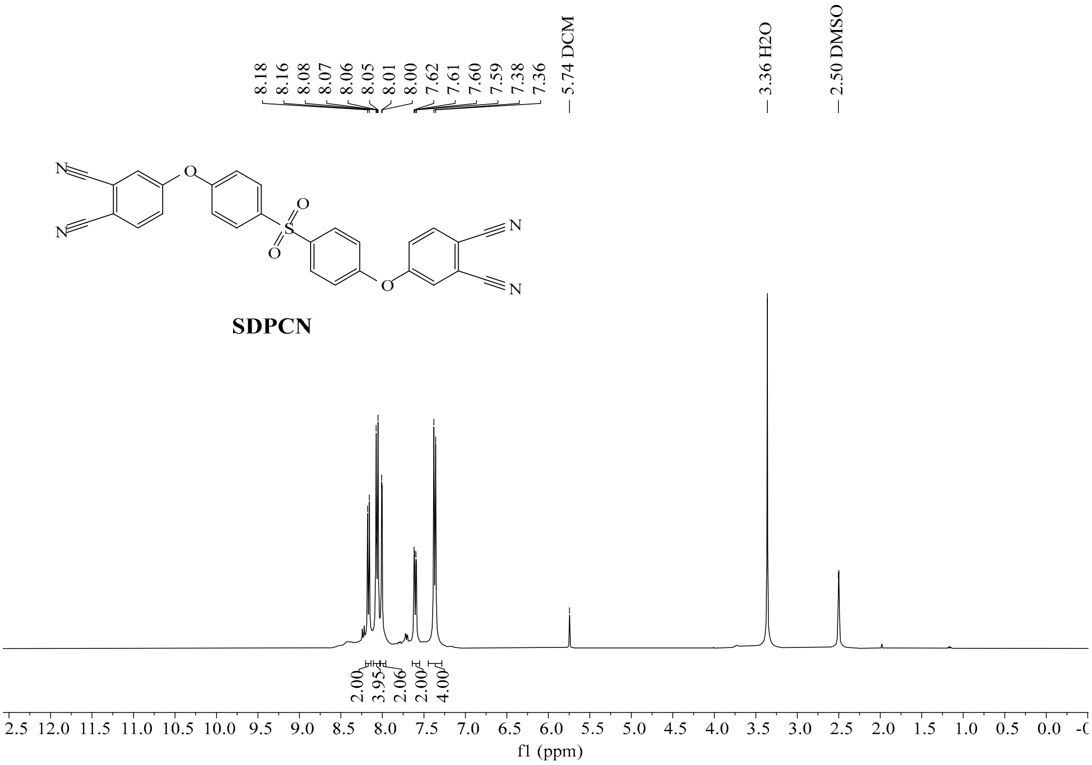

Figure S12. ¹H NMR spectrum of SDPCN in DMSO-*d6*

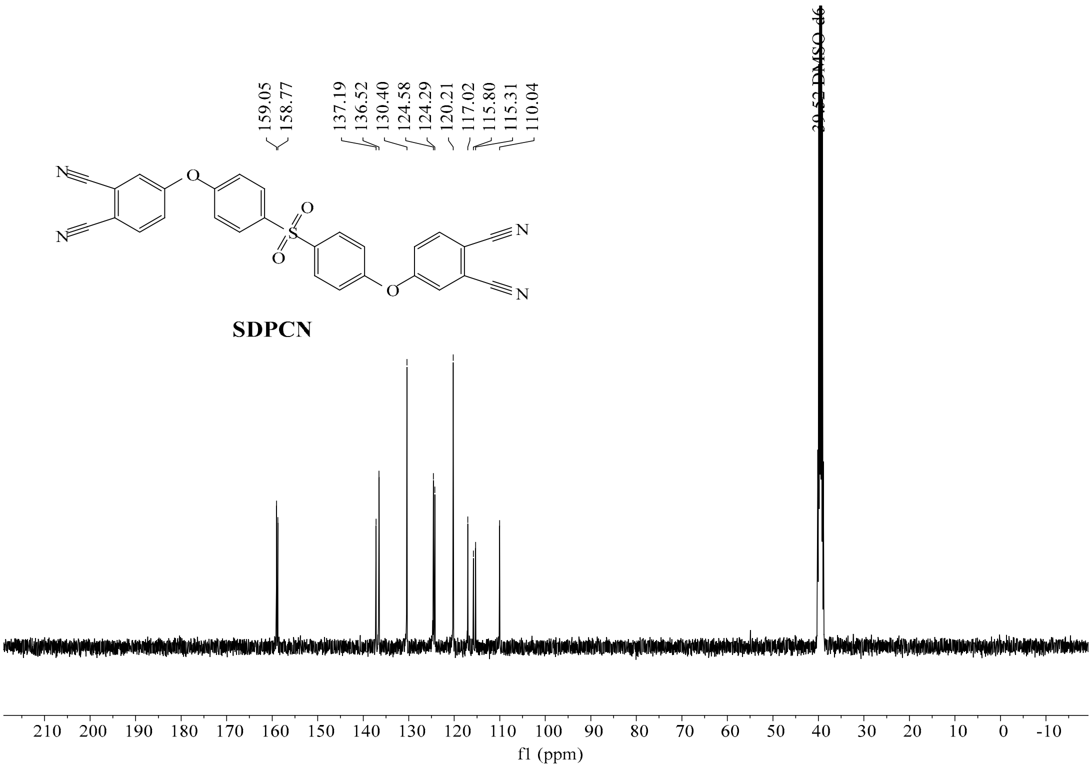

Figure S13. ¹³C NMR spectrum of SDPCN in DMSO-*d6*

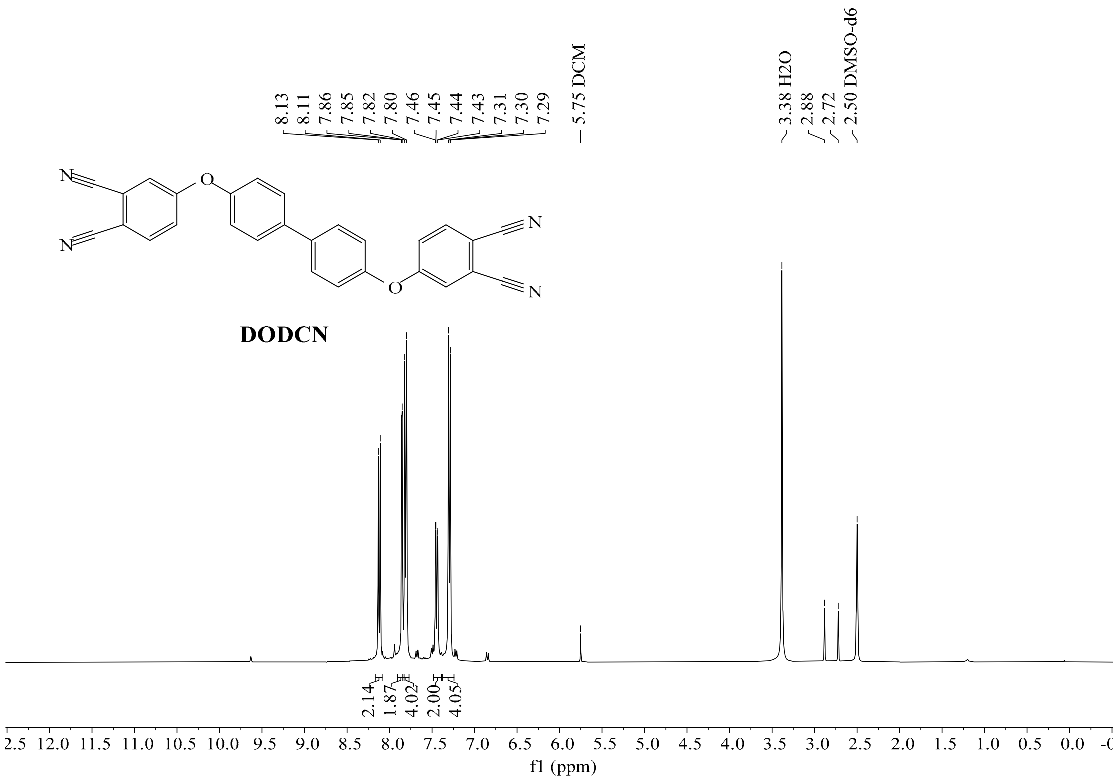

Figure S14. ^{1}H NMR spectrum of DODCN in DMSO-*d6*

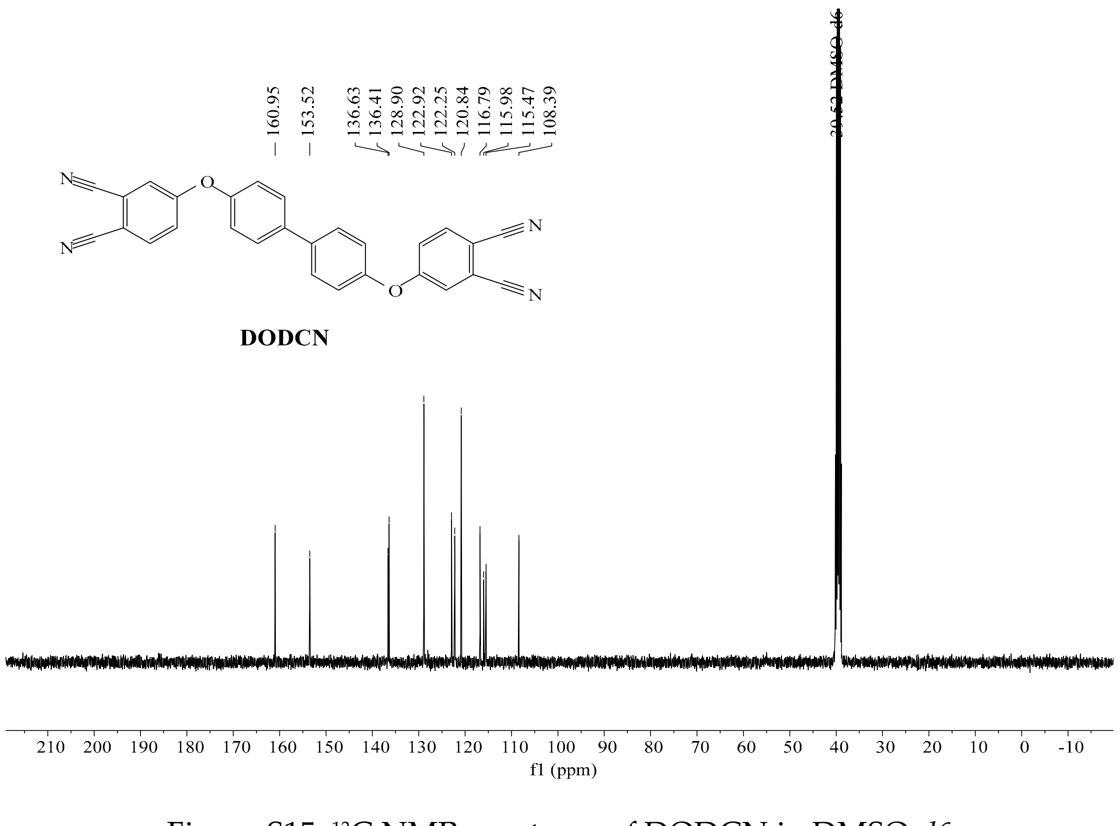

Figure S15. ^{13}C NMR spectrum of DODCN in DMSO-*d6*

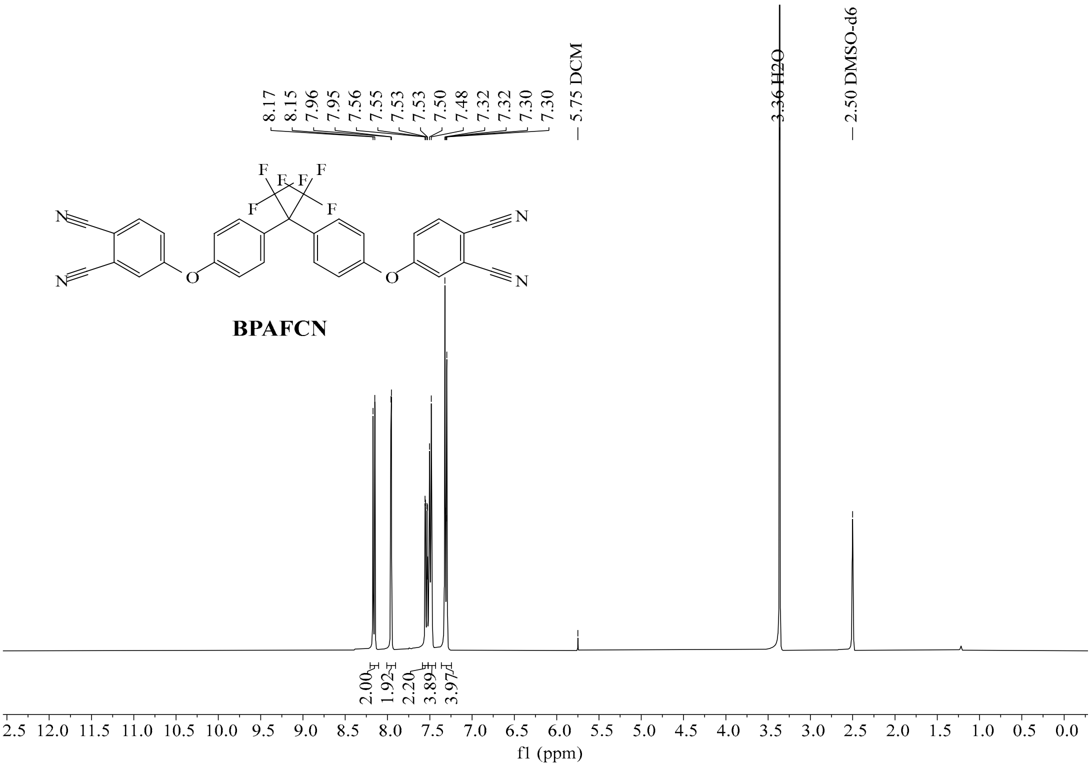

Figure S16. ¹H NMR spectrum of BPAFCN in DMSO-*d6*

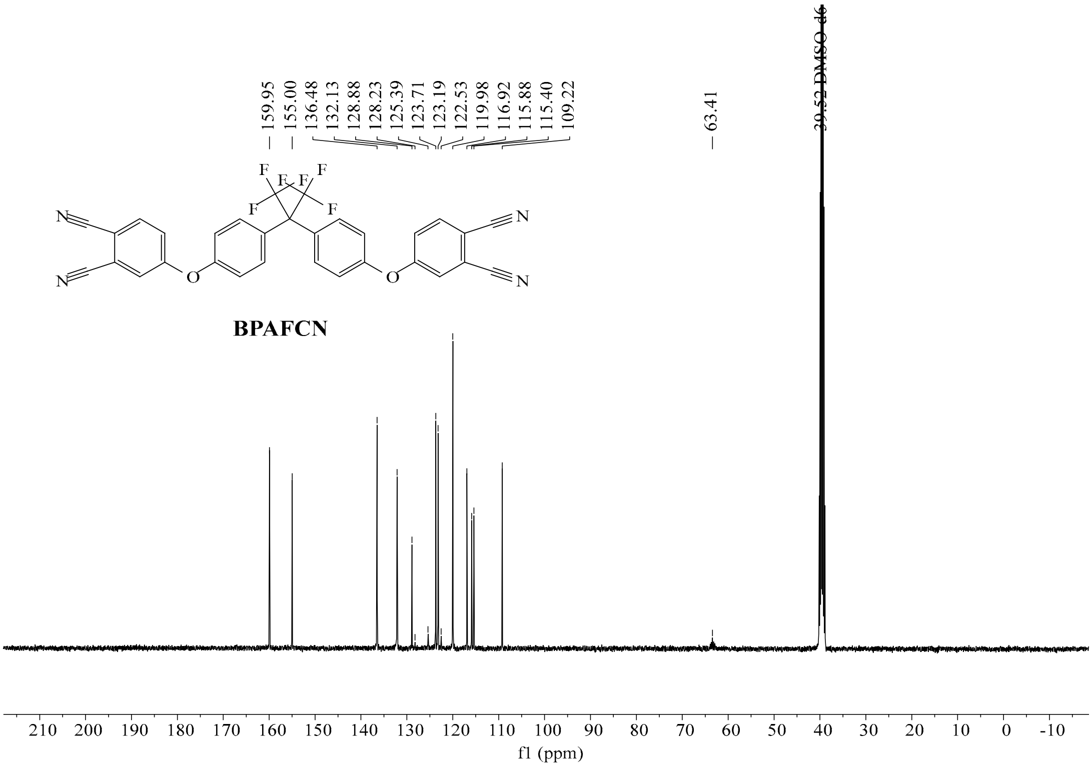

Figure S17. ¹³C NMR spectrum of BPAFCN in DMSO-*d6*

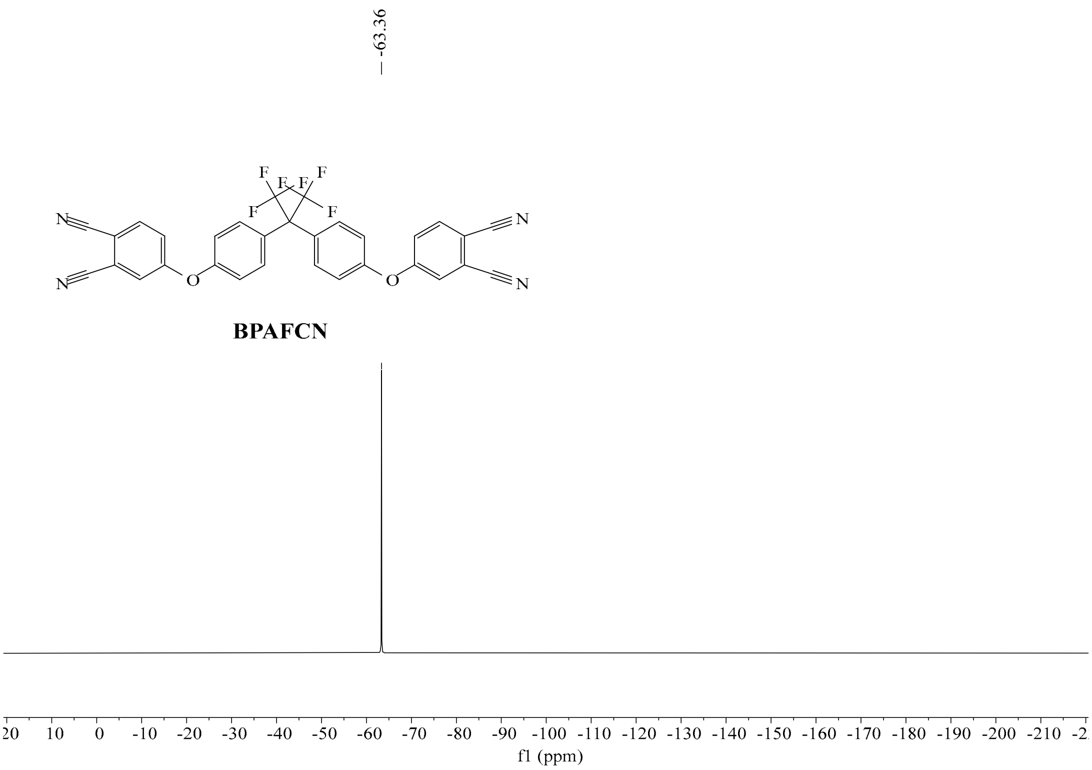

Figure S18. ^{19}F NMR spectrum of BPAFCN in DMSO-*d6*

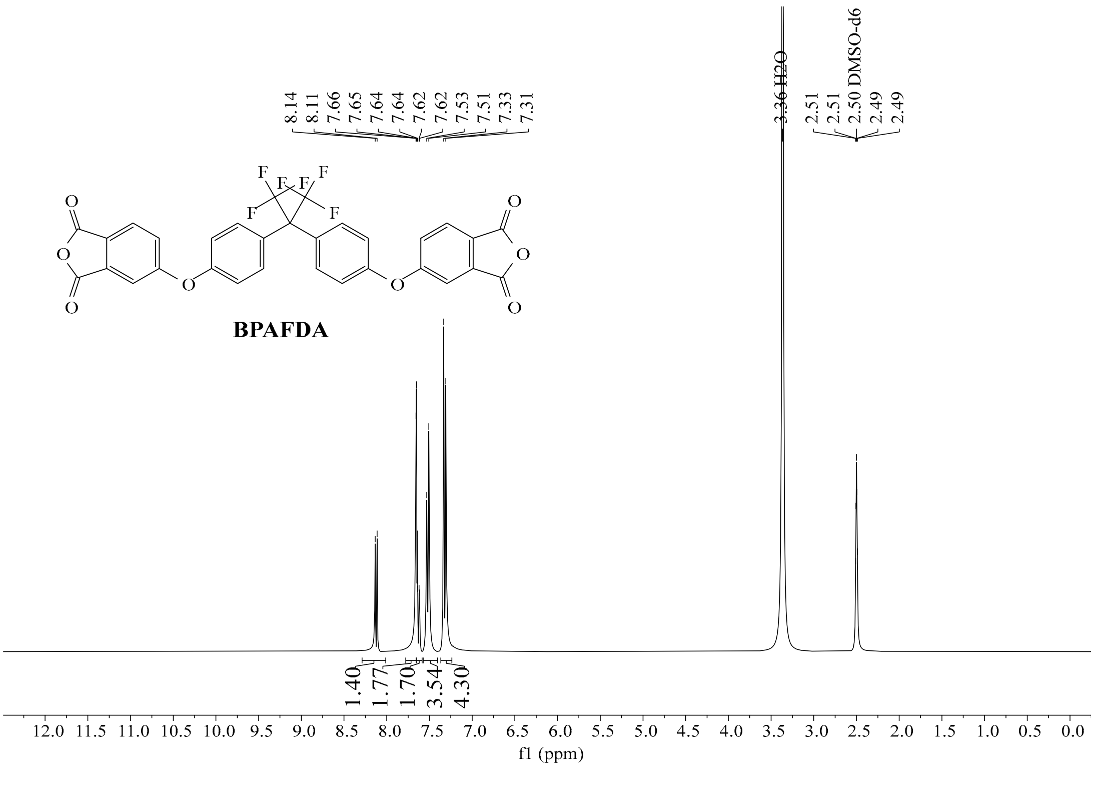

Figure S19. ^{1}H NMR spectrum of BPAFDA in DMSO-*d6*

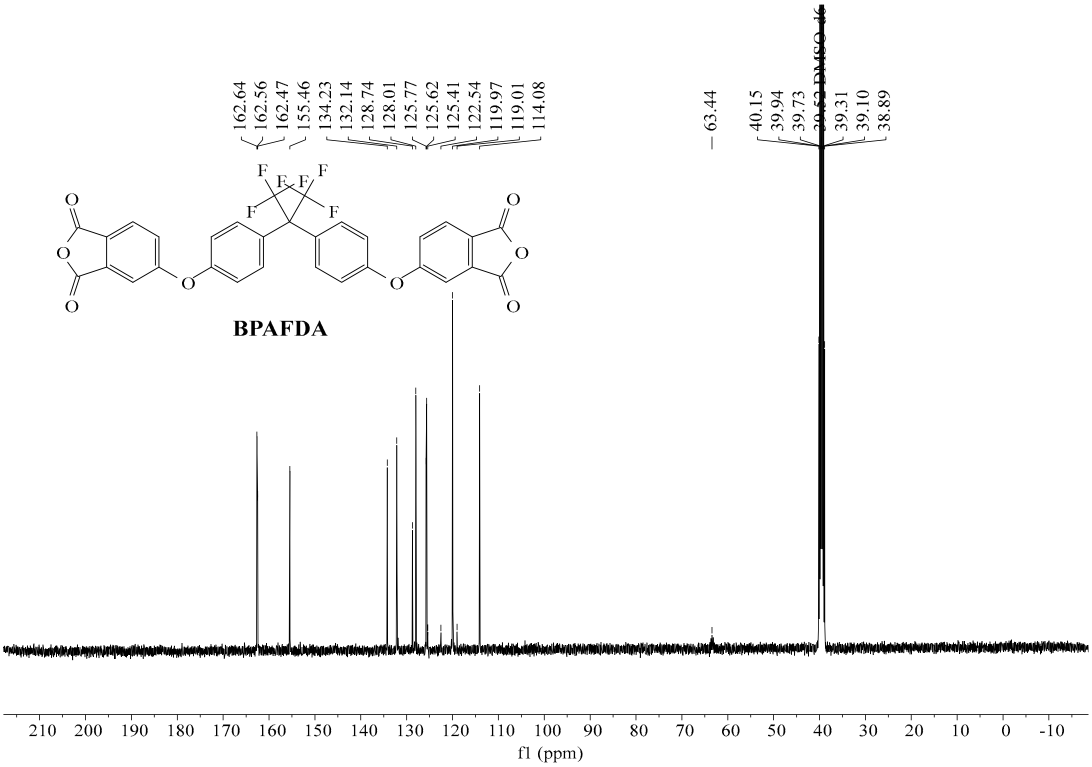

Figure S20. ^{13}C NMR spectrum of BPAFDA in DMSO-*d6*

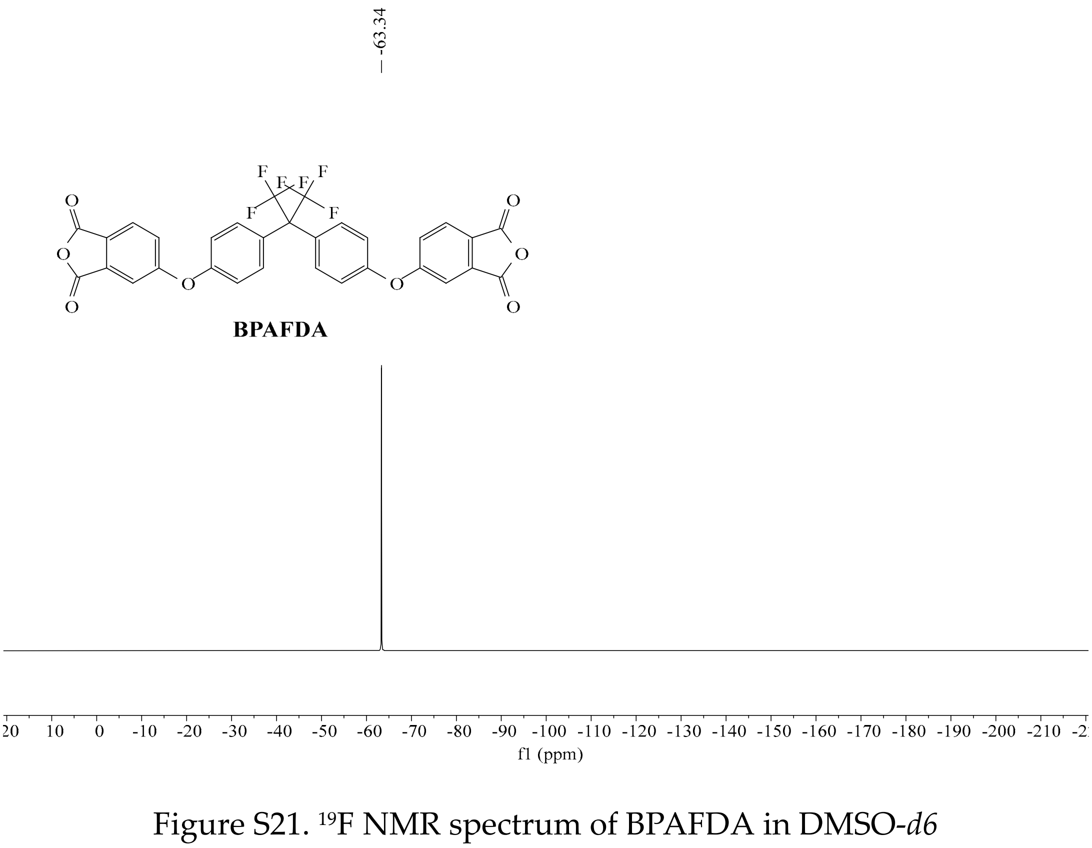

Figure S21. ^{19}F NMR spectrum of BPAFDA in DMSO-*d6*

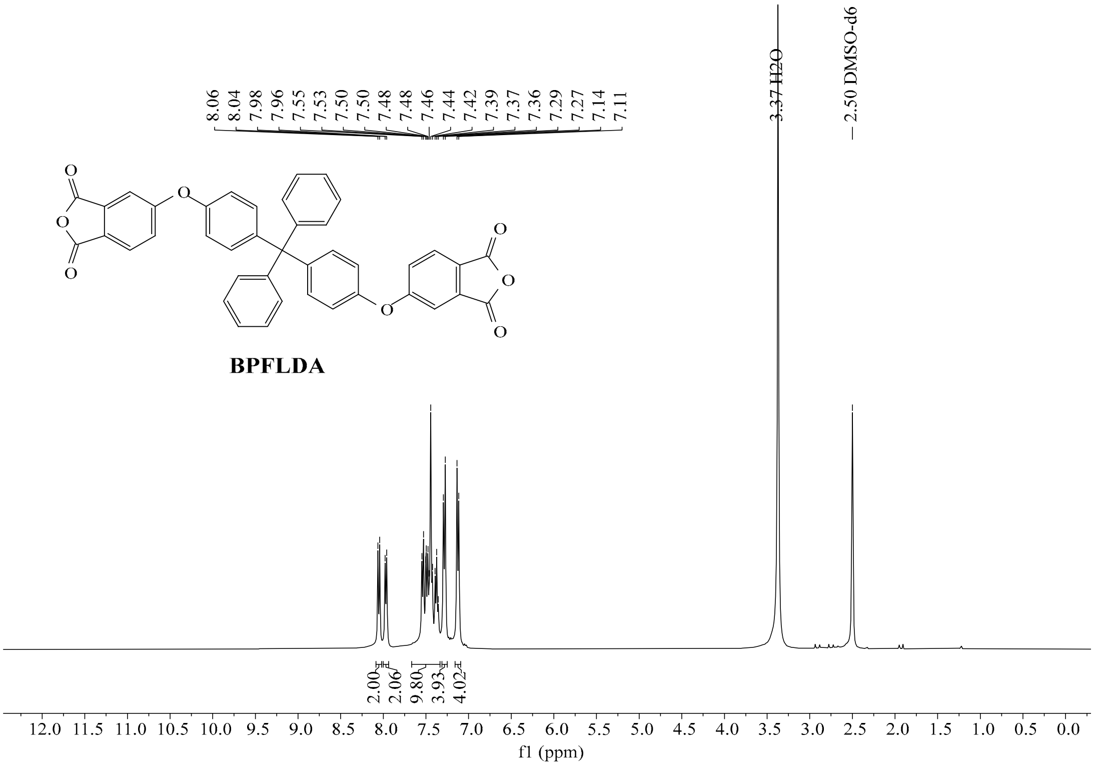

Figure S22. ^{1}H NMR spectrum of BPFLDA in DMSO-*d6*

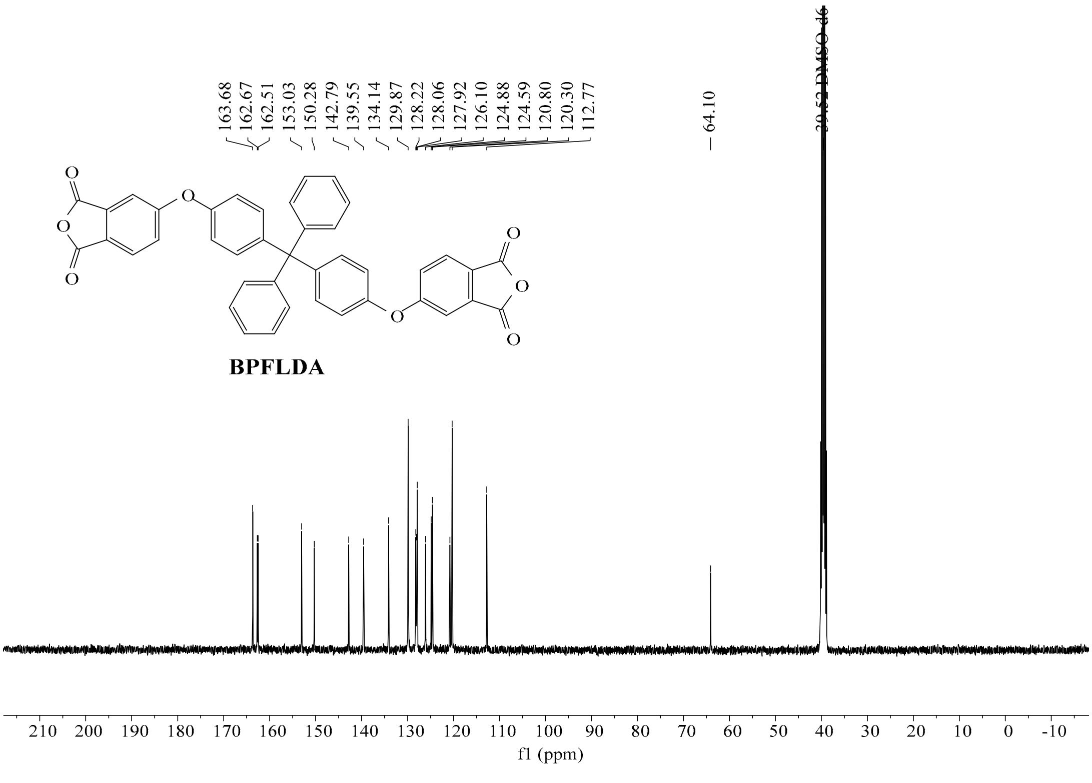

Figure S23. ^{13}C NMR spectrum of BPFLDA in DMSO-*d6*

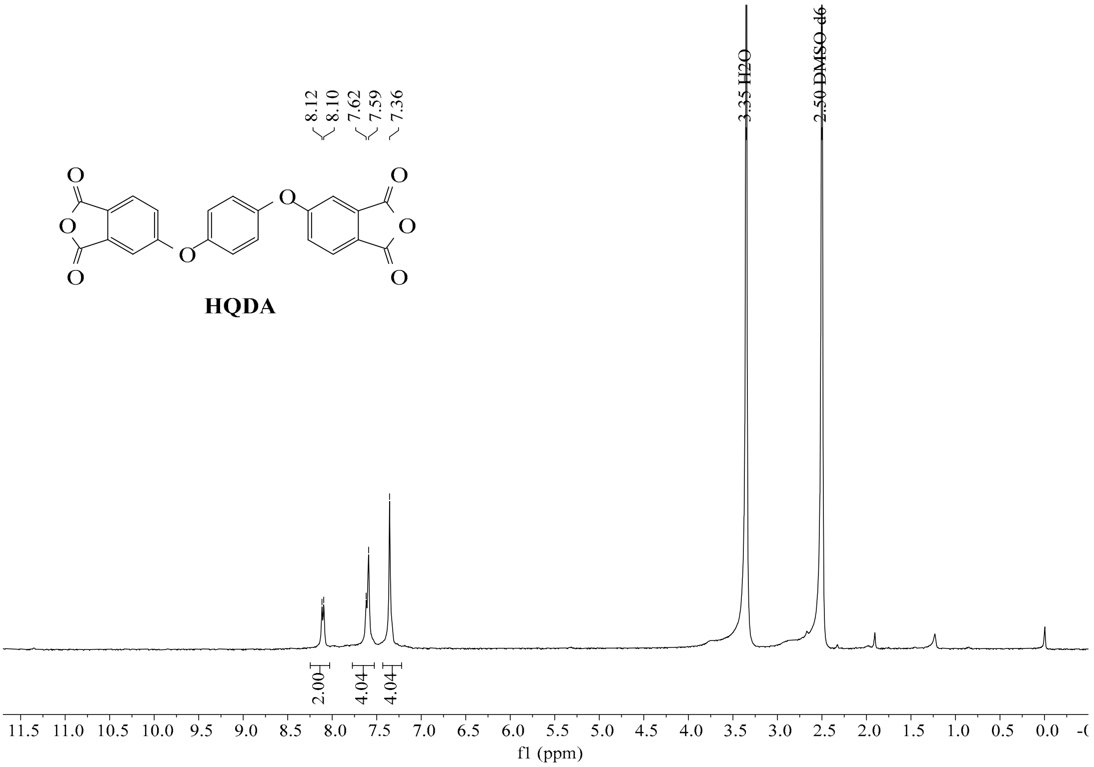

Figure S24. ^{1}H NMR spectrum of HQDA in DMSO-*d6*

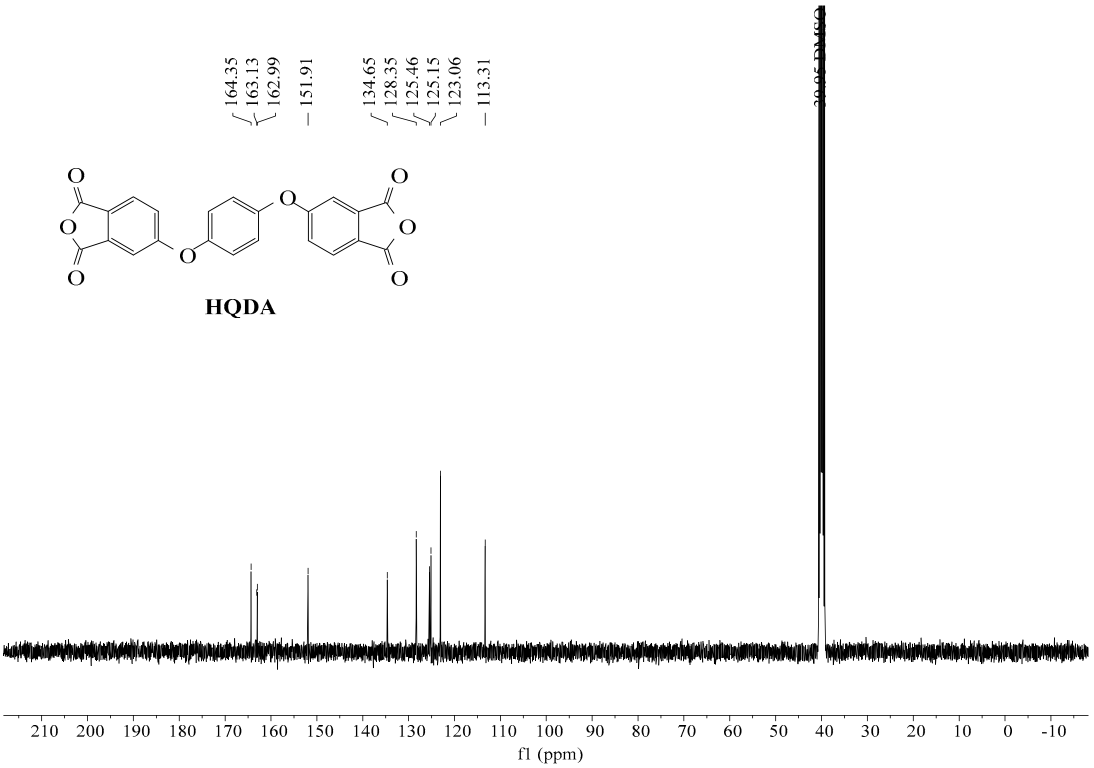

Figure S25. ^{13}C NMR spectrum of HQDA in DMSO-*d6*

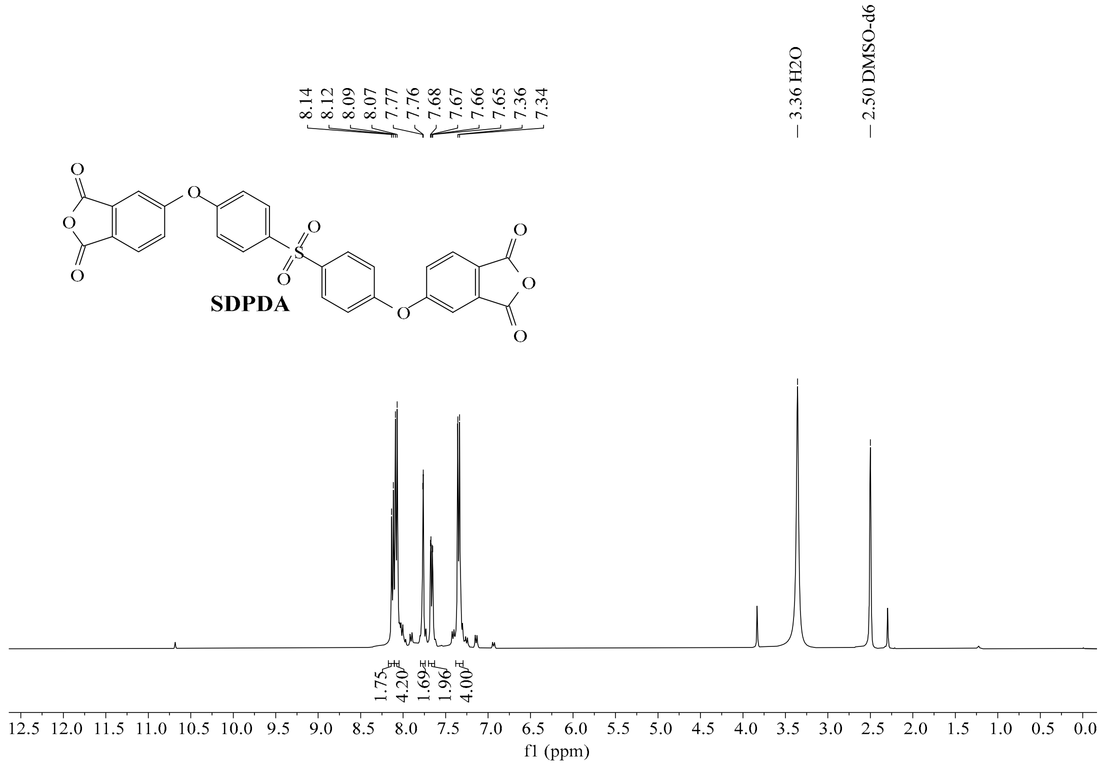

Figure S26. ^{1}H NMR spectrum of SDPDA in DMSO-*d6*

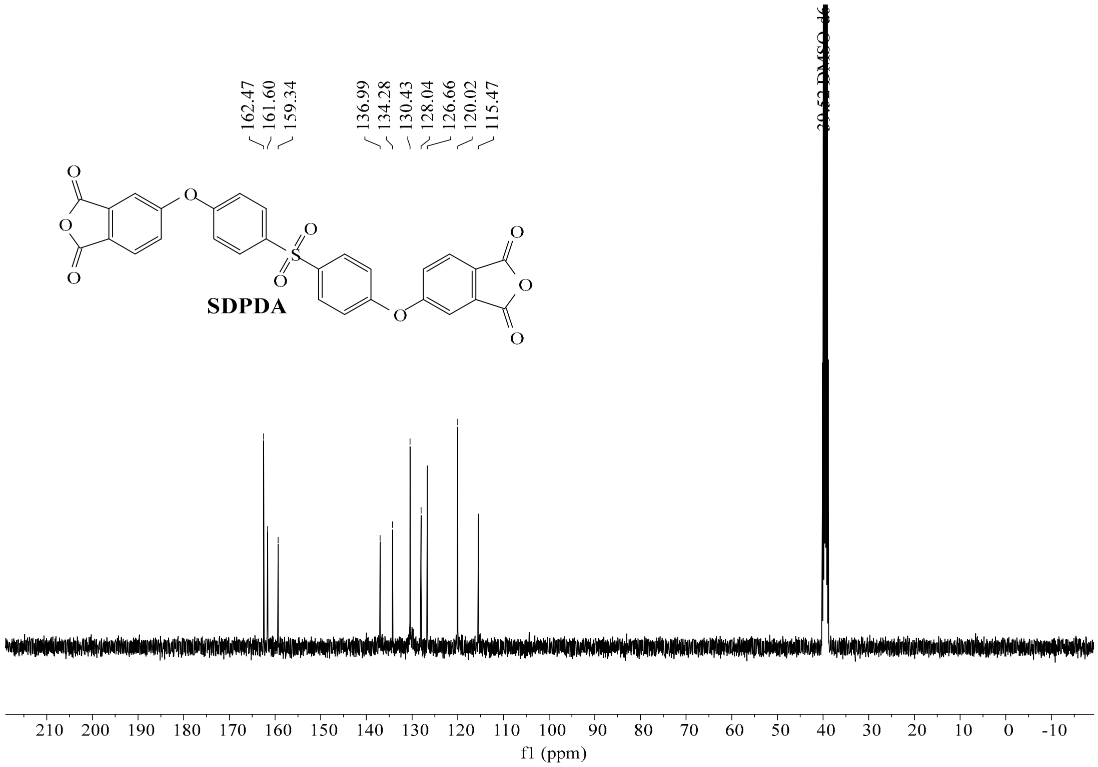

Figure S27. ^{13}C NMR spectrum of SDPDA in DMSO-*d6*

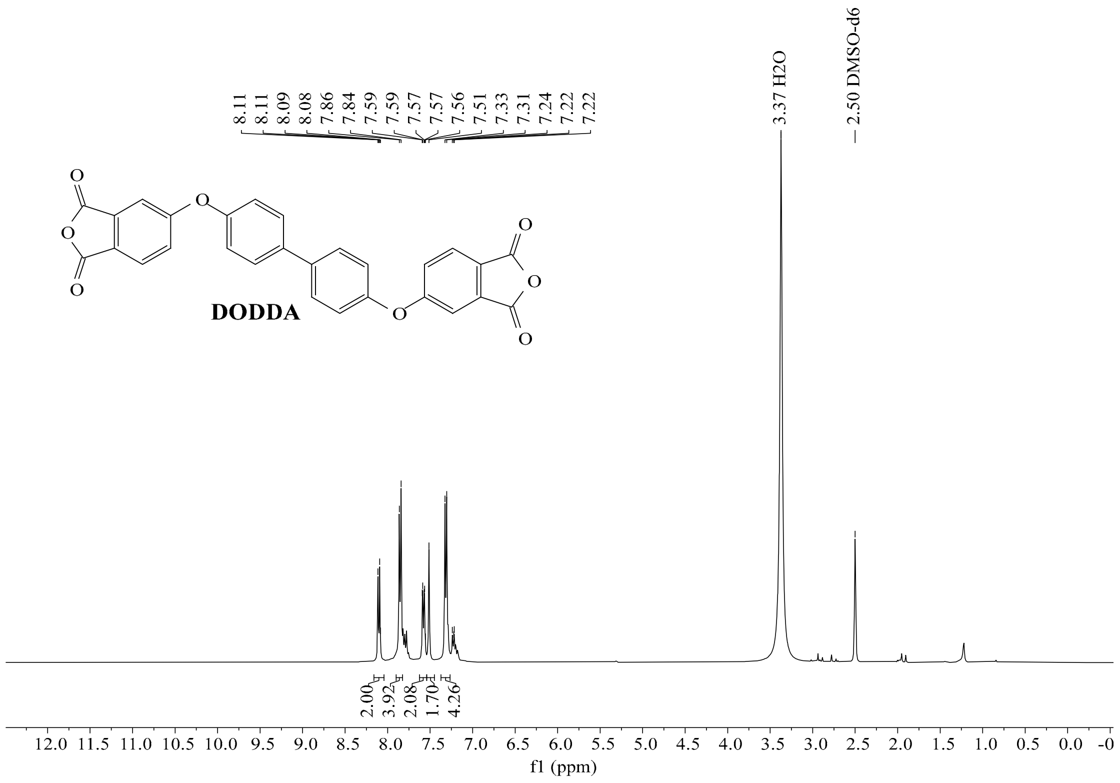

Figure S28. ^{1}H NMR spectrum of DODDA in DMSO-*d6*

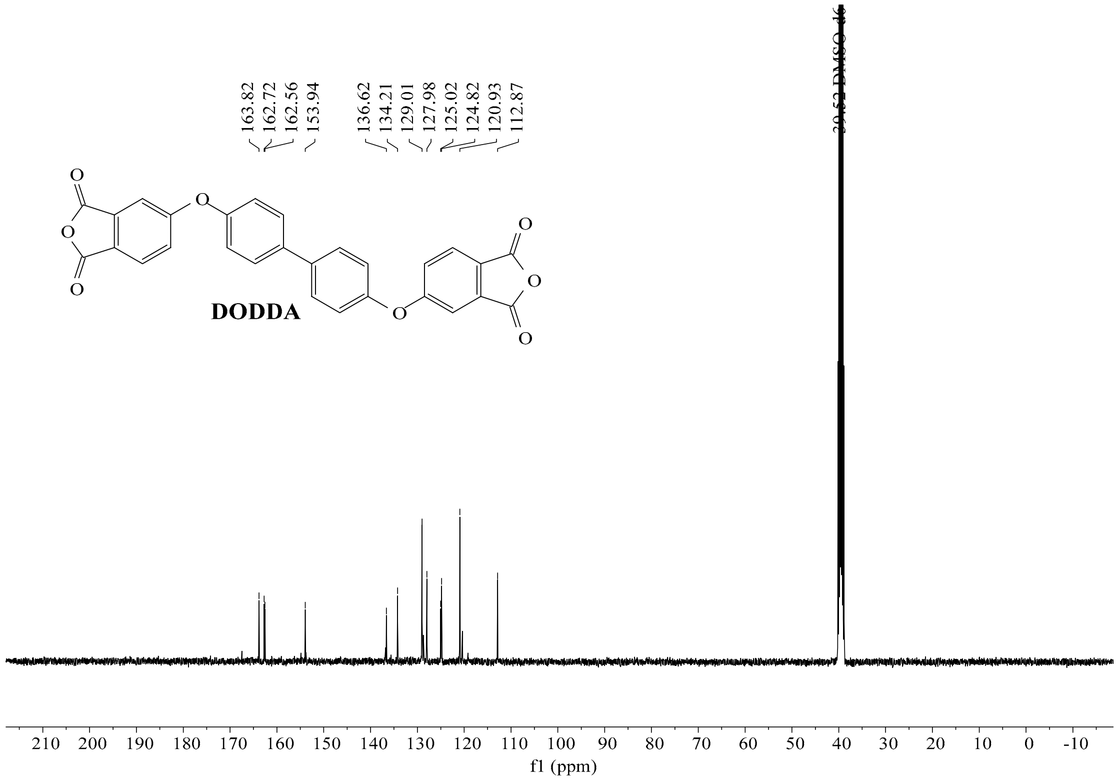

Figure S29. ^{13}C NMR spectrum of DODDA in DMSO-*d6*
